# Analysis of the Localization of Fluorescent PpROP1 and PpROP-GEF4 Fusion Proteins in Moss Protonemata Based on Genomic “Knock-In” and Estradiol-Titratable Expression

**DOI:** 10.3389/fpls.2019.00456

**Published:** 2019-04-12

**Authors:** Aude Le Bail, Sylwia Schulmeister, Pierre-François Perroud, Maria Ntefidou, Stefan A. Rensing, Benedikt Kost

**Affiliations:** ^1^Cell Biology, Department of Biology, Friedrich–Alexander University Erlangen–Nürnberg, Erlangen, Germany; ^2^Plant Cell Biology, Faculty of Biology, Philipps University of Marburg, Marburg, Germany

**Keywords:** XFP tagging, genomic knock-in, estradiol-inducible expression, *Physcomitrella patens*, ROP signaling

## Abstract

Tip growth of pollen tubes, root hairs, and apical cells of moss protonemata is controlled by ROP (Rho of plants) GTPases, which were shown to accumulate at the apical plasma membrane of these cells. However, most ROP localization patterns reported in the literature are based on fluorescent protein tagging and need to be interpreted with caution, as ROP fusion proteins were generally overexpressed at undefined levels, in many cases without assessing effects on tip growth. ROP-GEFs, important regulators of ROP activity, were also described to accumulate at the apical plasma membrane during tip growth. However, to date only the localization of fluorescent ROP-GEF fusion proteins strongly overexpressed using highly active promoters have been investigated. Here, the intracellular distributions of fluorescent PpROP1 and PpROP-GEF4 fusion proteins expressed at essentially endogenous levels in apical cells of *Physcomitrella patens* “knock-in” protonemata were analyzed. Whereas PpROP-GEF4 was found to associate with a small apical plasma membrane domain, PpROP1 expression was below the detection limit. Estradiol-titratable expression of a fluorescent PpROP1 fusion protein at the lowest detectable level, at which plant development was only marginally affected, was therefore employed to show that PpROP1 also accumulates at the apical plasma membrane, although within a substantially larger domain. Interestingly, RNA-Seq data indicated that the majority of all genes active in protonemata are expressed at lower levels than *PpROP1*, suggesting that estradiol-titratable expression may represent an important alternative to “knock-in” based analysis of the intracellular distribution of fluorescent fusion proteins in protonemal cells.

## Introduction

Unidirectional expansion (“tip growth”) of plant cells requires an elaborate, dynamic actin cytoskeleton and a highly active membrane trafficking system, which together mediate massive local secretion of cell wall material at the single growth site (Yalovsky et al., 2008). Protonemal filaments of the moss *Physcomitrella patens*, which can display either chloronemal or caulonemal characteristics, elongate based on tip growth of their apical cell (Menand et al., 2007). These filaments represent an excellent model for the investigation of molecular and cellular mechanisms underlying tip growth (Vidali and Bezanilla, 2012). A number of studies have shown that different actin associated proteins, such as profilin (Vidali et al., 2007), formins (Vidali et al., 2009), actin depolymerizing factor (Augustine et al., 2008, 2011), myosin XI (Vidali et al., 2010), as well as subunits of the Arp2/3 (Harries et al., 2005; Perroud and Quatrano, 2006; Finka et al., 2008) and the WAVE (Perroud and Quatrano, 2008) complexes, are essential for tip growth in *P. patens* protonemata. These proteins appear to be responsible for the maintenance of a dynamic actin cytoskeleton that is required for the directed transport of secretory vesicles toward the growth site at the tip (Bibeau et al., 2018).

Actin dynamics and membrane trafficking in plant cells are regulated and coordinated by Rac/ROP GTPases (hereafter named ROP GTPases), the plant representatives of the Rho family of small GTPases (Yalovsky et al., 2008). Extensive cell biological and genetic evidence demonstrates that ROP GTPases accumulate at the plasma membrane at the apex of root hairs and pollen tubes of vascular plants, where they play a key role in the control of tip growth (Kost, 2008). However, ROP localization was generally investigated in root hairs and pollen tubes either by immunolabeling of chemically fixed and/or permeabilized cells (Lin et al., 1996; Molendijk et al., 2001), which only ineffectively preserves the structural organization of tip-growing cells (He and Wetzstein, 1995; Doris and Steer, 1996), or based on overexpressing fluorescent fusion proteins at unknown levels (Kost et al., 1999; Gu et al., 2005). Available data concerning the intracellular distribution of these proteins during tip growth therefore need to be interpreted with caution. In recognition of this issue, the association of fluorescent ROP fusion proteins with the apical plasma membrane has recently begun to be investigated in pollen tubes overexpressing such proteins at low levels, at which tip growth was not or only moderately affected (Sun et al., 2015; Li et al., 2018).

ROP GTPases are membrane-associated as a consequence of posttranslational prenylation at the C-terminus (Sorek et al., 2011), and interact with downstream effectors specifically in the GTP bound conformation. Different upstream regulators interact with ROP GTPases to control their signaling function. GTPase-activating proteins (ROP-GAPs) inactivate the ROP GTPase signaling function by stimulating their low intrinsic GTPase activity. Guanine nucleotide dissociation inhibitors (ROP-GDIs) enable the translocation of ROP GTPases from the plasma membrane to the cytoplasm, where the two proteins form inactive heterodimers. Plant specific guanine nucleotide exchange factors (ROP-GEFs) activate the signaling function of ROP GTPases by promoting the exchange of GDP for GTP (Berken et al., 2005). ROP-GEFs generally appear to have a crucial function in the spatial control of ROP activity in response to extracellular signals (Zhang and McCormick, 2007; Chang et al., 2013). Consistent with such a function, GFP tagged *Medicago truncatula* and *Arabidopsis thaliana* ROP-GEFs were observed to accumulate at the plasma membrane at the tip of *M. truncatula* root hairs (Riely et al., 2011) and of tobacco pollen tubes (Gu et al., 2006), respectively. However, in both cell types GFP::ROP-GEF fusion proteins were overexpressed at high levels under the control of a strong promoter, which massively affected tip growth particularly in the analyzed tobacco pollen tubes. Furthermore, *A. thaliana* proteins may display non-physiological distribution patterns when heterologously expressed in these cells.

The *P. patens* genome has been shown to contain genes coding for homologs of all protein families involved in ROP signaling, including four *PpROP* genes encoding nearly identical proteins and six more diverse *PpROP-GEF* genes (Eklund et al., 2010). An essential function of *P. patens* ROPs in the control of tip growth has been demonstrated by simultaneously knocking-down all four ROPs expressed in this moss based on an RNA interference (RNAi) approach, which was found to alter actin dynamics and to completely block polarized cell expansion (Burkart et al., 2015). In addition, ROP or ROP-GEF overexpression has been shown to depolarize cell expansion at the tip of *P. patens* protonemata (Ito et al., 2014). In the same study, ROP accumulation at the plasma membrane of protonemal cells was demonstrated. However, the cells analyzed in these experiments displayed abnormal expansion and morphology because of high-level ROP overexpression.

Efficient incorporation of transgenes into the *P. patens* genome based on homologous recombination enables “knock-in” of cDNA sequences coding for fluorescent proteins precisely at the 5′ or 3′ end of selected target genes. This approach can be employed to generate reporter lines that express fluorescent proteins fused to the N- or C-terminus of selected target proteins. Fluorescent protein “knock-in” is potentially an excellent method to investigate protein localization in *P. patens*, as it may allow observation of the intracellular distribution of fluorescent fusion proteins that are expressed at endogenous levels under the control of the native genomic environment of the targeted genes. However, successful application of this strategy has only rarely been reported in the literature to date (e.g., Perroud and Quatrano, 2006; Hiwatashi et al., 2014; Miki et al., 2014; Sugita et al., 2014; Wiedemann et al., 2018), possibly because endogenous expression levels are often insufficient for effective microscopic imaging of fluorescent fusion proteins. Much more often, protein localization studies in *P. patens* have been performed based on stable overexpression of fluorescent fusion proteins under the control of strong, constitutively active promoters [rice actin promoter (Viaene et al., 2014); maize ubiquitin promoter (Vidali et al., 2010)] or of a heat shock inducible promoter (Ito et al., 2014). As discussed above, the results of such studies have to be interpreted with caution, as fluorescent fusion proteins often affect the structural organization of observed cells and display non-physiological intracellular distribution patterns when overexpressed at high levels (Crivat and Taraska, 2012; Stephan et al., 2014; Sun et al., 2015).

As an alternative to constitutive overexpression, an estradiol inducible gene expression system developed for flowering plants (Zuo et al., 2000) has been adapted for use in *P. patens* (Kubo et al., 2013). This system enables titration of transgene expression levels by applying estradiol at different concentrations. It is based on constitutive expression of a recombinant chimeric transcription factor called XVE, which is composed of the DNA-binding domain of the bacterial repressor LexA, the transcriptional activation domain of the viral protein VP16, and the regulatory region of the human estrogen receptor. Only in the presence of estradiol, the XVE transcription factor is capable of binding to an artificial promotor comprised of several copies of the LexA operator fused to a minimal CaMV35S promoter sequence, which results in RNA polymerase II recruitment and induction of downstream gene expression. In *P. patens*, constructs containing a constitutively active XVE expression cassette along with the artificial estradiol inducible promoter fused to cDNAs coding for fluorescent fusion proteins can be inserted into the “*P. patens* intergenic region 1” (PIG1) of the genome to standardize positional effects on transgene expression (Okano et al., 2009).

In the presented study, we pursued “knock-in” strategies to investigate in *P. patens* protonemata the intracellular distributions of YFP::PpROP1 and PpROP-GEF4::GFP fusion proteins expressed at essentially endogenous levels. PpROP-GEF4 displays the strongest and most specific expression at the transcript level of all *P. patens* ROP-GEFs in wild-type (WT) protonemata, and interacts with PpROP1 in yeast two-hybrid assays. Interestingly, our analysis of *PpROP-GEF4::GFP* “knock-in” protonemata established that PpROP-GEF4::GFP highly and specifically accumulates at the plasma membrane of apical cells within a small dome-shaped domain at the extreme tip. By contrast, YFP::PpROP1 was not detectable by confocal imaging in *YFP::PpROP1* “knock-in” protonemata. This fusion protein was therefore expressed under the control of a XVE-inducible, estradiol-titratable promoter at the lowest detectable level, at which growth and morphology of analyzed protonemata was not detectably affected during the first 5 days of plant regeneration from protoplasts in the presence of estradiol. Under these conditions YFP::PpROP1 was also found to accumulate at the plasma membrane within a dome-shaped domain at the extreme tip of apical protonemal cells, although this domain was substantially more extended than the one labeled by PpROP-GEF4::GFP. Furthermore, YFP::PpROP1 was observed to associate with the plasma membrane underlying the cross wall that separates apical cells from directly adjacent neighboring cells within protonemal filaments. Together, these results establish the intracellular distributions of two central regulators of the tip growth of apical protonemal cells. In addition, they demonstrate that estradiol-titratable expression represents an excellent alternative approach to investigate the intracellular distribution of proteins like PpROP1 that display endogenous expression levels too low for the successful application of “knock-in” strategies. Interestingly, global transcriptome analysis based on RNA sequencing revealed that *PpROP1* transcripts accumulate to relatively high levels in *P. patens* protonemata. Together with the scarcity of reports describing successful GFP-tagging using “knock-in” strategies, this indicates that many fluorescent fusion proteins may not be detectable by microscopic imaging when expressed at endogenous levels in *P. patens* and perhaps also in other plants.

## Materials and Methods

### “Knock-In” and Estradiol-Inducible Expression Constructs

A *PpROP-GEF4* GFP “knock-in” construct was generated based on the plasmid pPpGFP described by Eklund et al. (2010), which contains a promoter-less *smRS-GFP-TNOS1* GFP tagging sequence [smRS-GFP; (Davis and Vierstra, 1998)] along with a constitutive *P35S-NPTII-T35S* expression cassette conferring kanamycin resistance (NptII). A genomic fragment starting immediately after the STOP codon of the *PpROP-GEF4* gene, which was amplified using the primers SLU440 and SLU441, was inserted into pPpGFP downstream of the *P35S-NPTII-T35S* expression cassette between unique *Not*I and *Xho*I sites. In addition, into the single *Bam*HI site of the resulting construct, a second genomic fragment amplified using the primers SLU438 and SLU444, which ends directly upstream of the STOP codon of the *PpROP-GEF4* gene, was inserted creating a translation fusion between the 3′ end of this gene and the *smRS-GFP-TNOS1* GFP tagging sequence (see Supplementary Figure S1).

To generate a *PpROP1* YFP “knock-in” construct, a genomic fragment ending immediately upstream of the START codon of the *PpROP1* gene, which was amplified using the primers FAU23 and FAU24, was cloned in pCR-BluntII-TOPO (Thermo Fisher Scientific; Waltham, MA, United States). Into the unique *Pac*I site of the resulting construct, a *YFP* cDNA (eYFP; BD Biosciences-Clontech; San Jose, CA, United States) amplified using the primers SLU433 and SLU434 was inserted downstream of the genomic *PpROP1* fragment to generate an intermediate construct. In addition, into the single *Eco*RV site of this intermediate construct, a second genomic fragment amplified using the primers FAU81 and FAU82, which starts exactly at the START codon of the *PpROP1* gene, was inserted creating a translational fusion between the 5′ end of this gene and 3′ end of the *YFP* cDNA (see Supplementary Figure S2).

A *PpROP1* 3xVENUS “knock-in” construct was generated using a similar strategy as described in the previous paragraph. First, a *3xVENUS* cDNA amplify from the plasmid pDR5rev::3XVENUS-N7 (Heisler et al., 2005) using the primers FAU-A56 and FAU-A57 was cloned in pCR-BluntII-TOPO. Between the unique *Cla*I and *Kpn*I sites of the resulting construct, a genomic fragment ending immediately upstream of the START codon of the *PpROP1* gene, which was amplified using the primers FAU-A81 and FAU-A104, was inserted upstream of the *3xVENUS* cDNA to generate an intermediate construct. Finally, between the unique *Eco*RV and *Not*I sites of this intermediate construct, a second genomic fragment amplified using the primers FAU-A82 and FAU-A83, which starts exactly at the START codon of the *PpROP1* gene, was inserted creating a translational fusion between the 5′ end of this gene and 3′ end of the *3xVENUS* cDNA (see Supplementary Figure S2).

The *YFP::PpROP1* and *3xVENUS::PpROP1* “knock-in” constructs were co-transformed with a plasmid designed to target an expression cassette conferring constitutive kanamycin resistance into the intergenic region Pp108B (Schaefer and Zrÿd, 1997). To generate this plasmid, pMP1432^1^ was restricted with *Bam*HI and *Pac*I to remove a heat shock promoter as well as gateway cassettes. Through blunting and religation of the two ends of the resulting linearized construct, a plasmid containing just a kanamycin resistance expression cassette flanked by Pp108B targeting sequences was created.

To develop a construct enabling estradiol inducible YFP::ROP1 expression, the *PpROP1* coding sequences was PCR amplified from *P. patens* total cDNA using the primers SLU32 and SLU33, and inserted between the unique *Xho*I and *Apa*I sites of pCMAK1 (Hiwatashi et al., 2008). Into the single *Xho*I site of the resulting plasmid, a cDNA encoding YFP fused at the C-terminus to a 5x Gly-Ala (5xGA) linker, which was PCR amplified from a *YFP::5xGA::NtRac5* construct (Sun et al., 2015) using the primers SLU70 and SLU71, was inserted to generate a plasmid containing a *YFP::5xGA::PpROP1* cDNA. This cDNA was PCR amplified using the primers FAU-A78 and FAU-A79, cloned into pENTR topo (Thermo Fisher Scientific; Waltham, MA, United States) and from there transferred via a “gateway” LR reaction (Thermo Fisher Scientific; Waltham, MA, United States) to pPGX8 (Kubo et al., 2013) such that it was inserted into this plasmid downstream of an artificial XVE responsive promoter. In addition to the XVE dependent *YFP::5xGA::PpROP1* expression cassette thus generated, the final construct also contained the following pPGX8 derived sequences: (a) a cassette conferring constitutive expression of the estradiol-inducible chimeric transcription factor XVE, (b) a constitutive *P35S-HygR-T35S* hygromycin resistance expression cassette, and (c) genomic PIG1bR and PIG1bL targeting sequences flanking the three described expression cassettes, which enable the insertion of these cassettes into the intergenic region 1 of the *P. patens* genome (PIG1) (Okano et al., 2009) based on homologous recombination (see Supplementary Figure S3).

The sequences of all PCR primers used to generate the constructs described above in this section are listed in Supplementary Table S1. All PCR amplified fragments and junctions between ligated fragments were confirmed by Sanger sequencing.

### Tissue Culture

All *P. patens* (ecotype Gransden) cultures were grown axenically in 9 cm plastic Petri dishes at 25°C under continuous white light with an intensity of 50 μmol m^-2^ s^-1^. All plant growth analyses (Figure 4, 10) were performed with plants regenerated from single protoplasts on solid culture media. During the first 7 days, small regenerating protonemata were kept on cellophane disks (AA Packaging, Preston, United Kingdom) covering the surface of the culture medium to facilitate transfer between different media. To determine plant area as well as length of the first subapical cell after 5 days, plants were first grown for 2 days on PRMB medium (6% mannitol and 10 mM of CaCl_2_ in BCDA medium) and subsequently transferred onto BCDA medium (Cove et al., 2009) for another 3 days. To measure plant area after 5 weeks, plants were treated as described in the previous sentence for the first 5 days, kept for two more days on BCDA medium, and finally removed from the cellophane disks and individually transferred onto BCD medium (Cove et al., 2009) for another 4 weeks. Analyses of protonemata based on fluorescence microscopy, quantitative PCR and immunoblotting were performed using 7-day-old plants regenerated from single protoplasts on solid culture medium, which had been grown first for 2 days on PRMB medium followed by another 5 days on BCDA medium. Estradiol was added both to the PRMB medium as well as to the BCDA medium as required. Leafy shoots without rhizoids used for qPCR analysis were removed from 5-week-old plants grown as described above.

### Protoplast Preparation and Transformation

For protoplast preparation, 6 to 7-day-old protonemata grown on BCDA from ground tissue were harvested, incubated in 0.5% driselase and 8.5% mannitol for 1 h at room temperature (RT) and subsequently filtered through a 100 μm mesh. The flow through containing protoplasts in suspension was centrifuged for 5 min at 100 *g*. Protoplasts collected in the pellet were washed twice with protoplast wash solution (8.5% mannitol and 10 mM CaCl_2_) and finally resuspended in protoplast wash solution to obtain a concentration of 0.1 million protoplasts per ml. The resulting protoplast suspension was spread on plates containing solid PRMB medium to initiate plant regeneration (1 ml/plate).

To generate transformed plants, a protocol described by Cove et al. (2009) was adapted. Protoplasts prepared and washed twice as described in the previous paragraph were resuspended in MMM solution (9.1% mannitol, 0.1% MES pH 7.6, and 15 mM MgCl_2_) at a concentration of 1.6 × 10^6^ protoplasts per ml. Three hundred microliters of the resulting protoplast suspension were mixed with 300 μl PEG solution (40% PEG 6000, 7% mannitol and 100 mM Ca[NO_3_]_2_), supplemented with 15 μg linearized plasmid DNA and finally heat-shocked for 5 min at 45°C. After a 10 min recovery period at RT, the protoplast suspension was progressively diluted by slowly adding five times 300 μl and five times 1 ml protoplast wash solution over a period of 30 min. Subsequently, protoplasts were pelleted by centrifugation and resuspended in 2 ml protoplast wash solution. After the addition of 3 ml PRMT medium (0.4% agar, 10 mM CaCl_2_, and 8% mannitol in BCDA medium), protoplasts were plated on four 9 cm Petri dishes containing solid PRMB medium (6% mannitol and 10 mM of CaCl_2_ in BCDA medium) overlaid with a cellophane disk. After 7 days, cellophane disks covered with regenerating plants were transferred for 1 week onto solid BCDA medium supplemented with an antibiotic (20 mg/l G418 or 30 mg/l hygromycin B), for another week onto antibiotic free BCDA medium and finally for an additional week back onto antibiotic-containing BCDA medium. Following this second round of antibiotic selection, surviving plants were removed from the cellophane disks and individually transferred in triplicates on antibiotic-free solid BCDA medium and grown for 10 days before DNA was extracted for PCR based genotyping.

### PCR Based Genotyping Including gDNA Extraction

Genomic DNA extraction was performed at RT (all steps required to obtain the final DNA solution) as described by Cove et al. (2009) with slight modifications. Briefly, plants transformed as described in the previous paragraph (one of the clonal triplicates generated) were homogenized in 200 μl water containing six glass beads (1 mm diameter) using a Tissuelyser II (Qiagen, Hilden, Germany). After the addition of 250 μl extraction buffer (0.36 M Tris pH 9, 0.72 M LiCl, 45 mM EDTA pH 8 and 1.8% SDS) the lysate was centrifuged for 10 min at 17000 *g* at RT. The supernatant was transferred to a new tube and 1 volume isopropanol was added to induce DNA precipitation. Precipitated DNA was pelleted by 10 min centrifugation at 17000 *g* and washed with 70% ethanol, dried and resuspended in TE buffer (10 mM Tris pH 8, 1 mM EDTA). One microliter of the resulting DNA solution and OneTaq DNA polymerase (New England BioLabs, Ipswich, MA, United States) were employed to amplify specific PCR fragments indicating correct transgene integration into target sites (PCR based genotyping). The sequences of all primers used for genotyping as indicated in Supplementary Figures S1–S3 are listed in Supplementary Table S1.

### Southern Blotting Including gDNA Extraction

For the Southern blotting, genomic DNA was extracted using the Nucleon PhytoPure kit (GE Healthcare, Chicago, IL, United States) from 7-day-old protonemata grown from ground tissue, which were harvested from 4 BCDA plates. Southern blotting was performed as described by Perroud and Quatrano (2008). To characterize transgene insertion in the *YFP-PpROP1^ind^* line, genomic DNA was digested with *Nco*I and hybridized with a probe recognizing the XVE coding sequence.

### Real-Time qPCR

**FIGURE 1:**
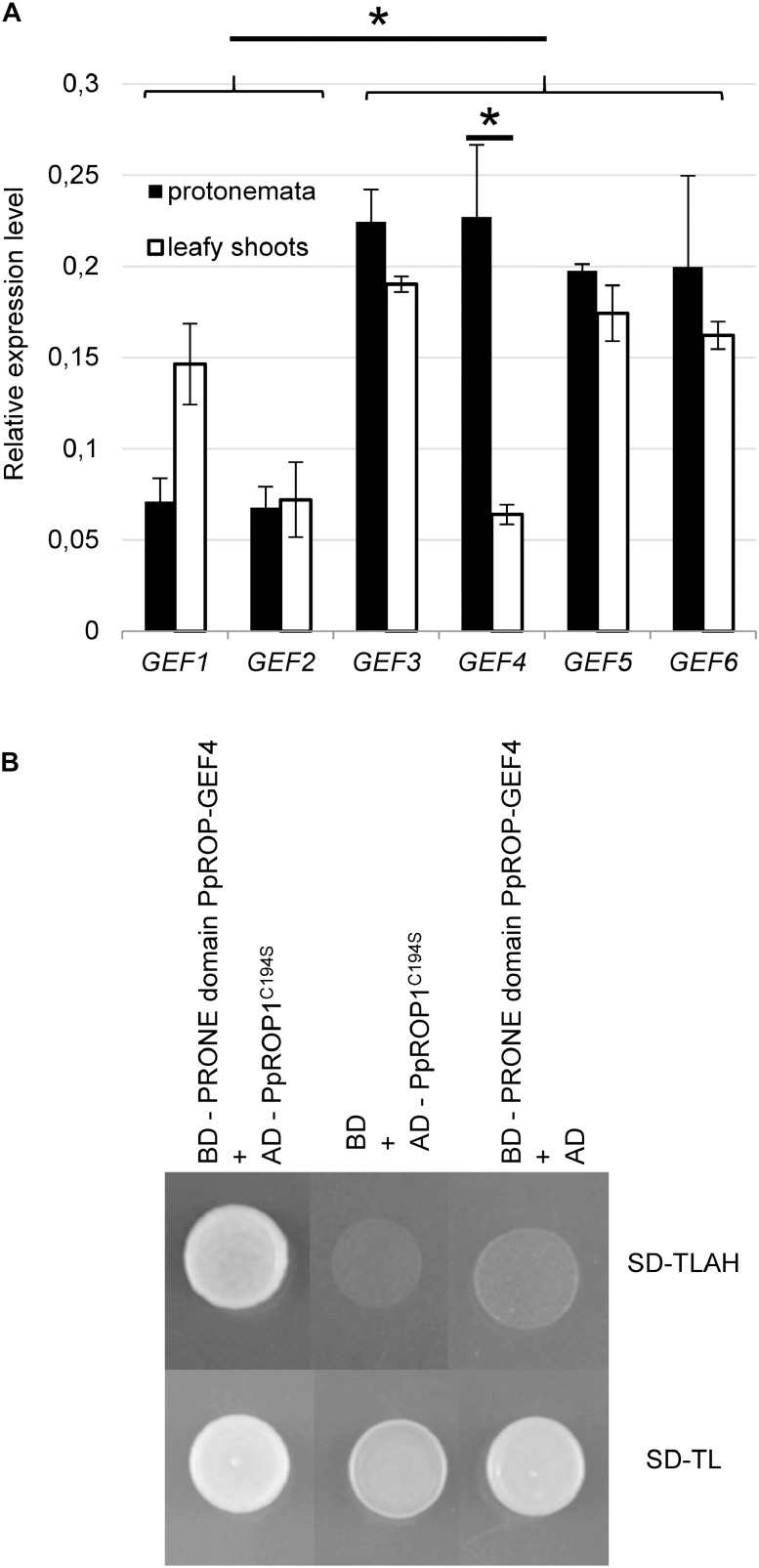
PpROP-GEF4 is preferentially expressed in protonemata and interacts with PpROP1 in yeast two-hybrid assays. **(A)** Comparative analysis by quantitative PCR (qPCR) of transcript levels of all 6 *P. patens PpROP-GEF* genes (*PpROP-GEF1-6*; indicated as *GEF1*-*GEF6*) in protonemata and leafy shoots. Relative expression levels are indicated, which represent absolute *PpROP-GEF* transcript levels normalized relative to the absolute transcript level of the reference gene *PpACTIN5*. The statistical significance of differences between all data sets was assessed using ANOVA (Bonferroni-Holm, one factor). ^∗^*P* < 0.05: statistically significant differences relevant in the context of this study (stronger expression of *PpROP-GEF3-6* than *PpROP-GEF1* and *2* in protonemata, stronger expression of *PpROP-GEF4* in protonemata than in leafy shoots). Error bars: standard deviation. **(B)** Yeast transformants co-expressing the catalytically active PRONE domain of PpROP-GEF4 fused to the DNA binding domain of the GAL4 transcription factor (BD) together with PpROP1 fused to the GAL4 activation domain (AD) plated on synthetic defined medium without tryptophan and leucine (SD-TL), or without tryptophan, leucine, adenine, and histidine (SD-TLAH). The PpROP1 bait protein carried a point mutation that enhances nuclear import by preventing posttranslational prenylation (C194S). Serving as negative controls were transformants co-expressing the PpROP-GEF4 PRONE domain bait protein with just the AD, or the PpROP1 prey protein with just the BD. Growth on SD-TLAH indicated two-hybrid interaction between the PpROP-GEF4 PRONE domain and PpROP1.

**FIGURE 2:**
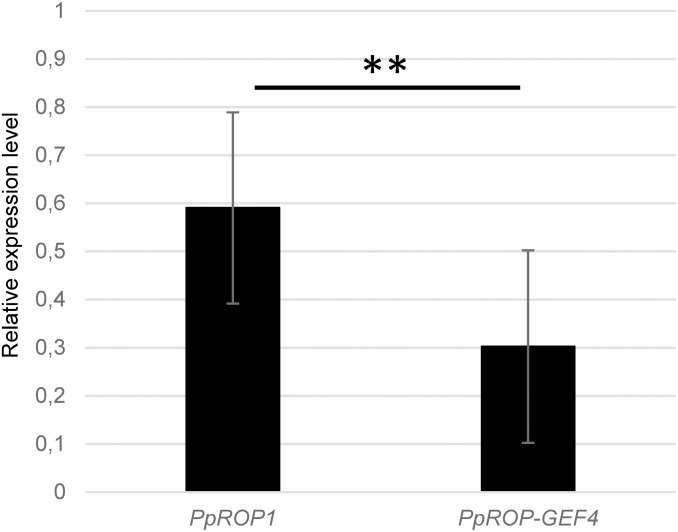
*PpROP1* displays somewhat higher transcript levels in protonemata than *PpROP-PpGEF4*. *PpROP1* and *PpROP-GEF4* expression levels in WT protonemata as determined by qPCR. Relative expression levels are indicated, which represent absolute *PpROP1* and *PpROP-GEF4* transcript levels normalized relative to the absolute transcript level of the reference gene *PpUbC-E2*. The statistical significance of differences between the two data sets was assessed using a Student’s *t*-test (two tailed, type II). ^∗∗^*P* < 0.01. Error bars: standard deviation.

**FIGURE 3:**
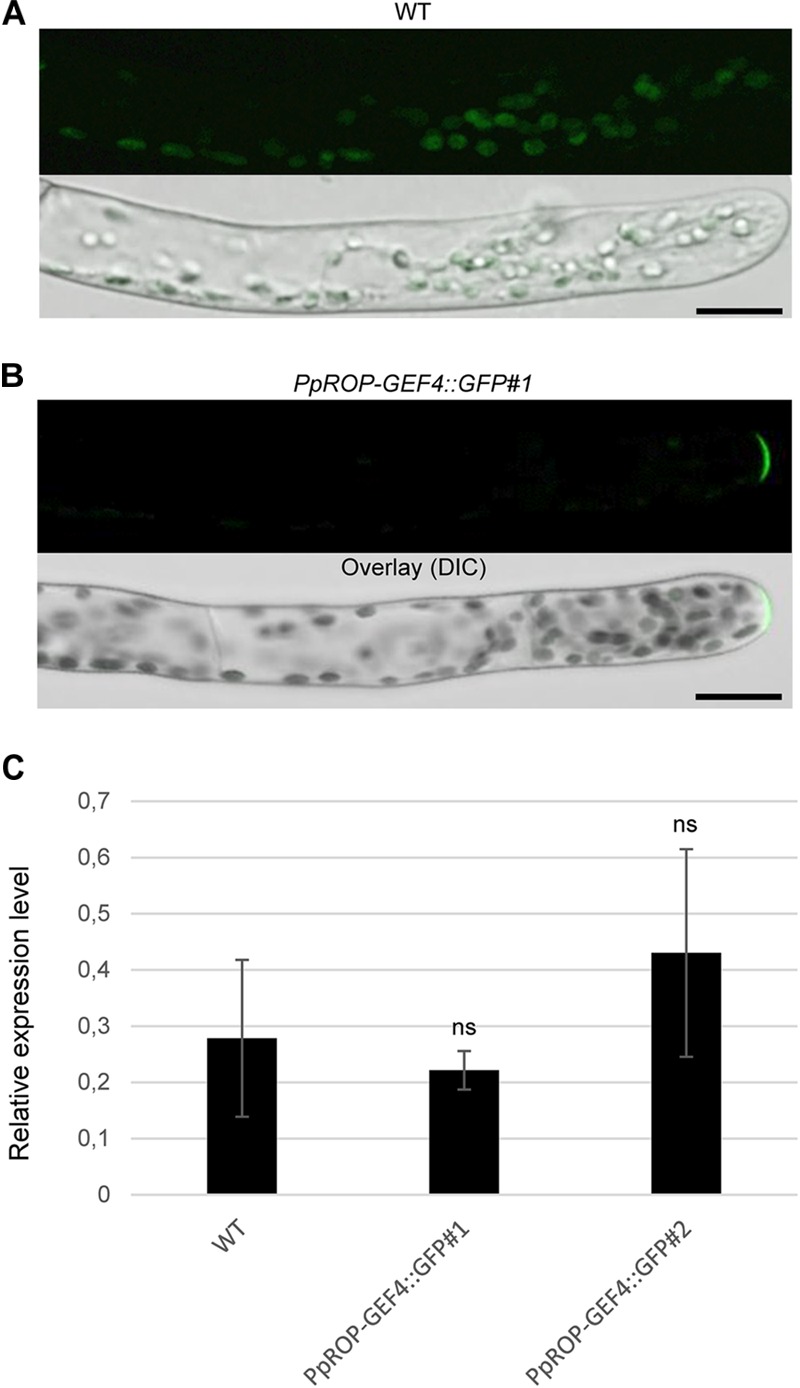
Analysis of the intracellular PpROP-GEF4::GFP distribution in apical cells of *PpROP-GEF4::GFP* “knock-in” protonemata. **(A,B)** Medial confocal optical sections through the tips of wild type (WT) **(A)** or *PpROP-GEF4::GFP#1* **(B)** protonemal filaments (upper panels). Only weak background chlorophyll autofluorescence was visible in WT protonemata (*n* = 15). All apical *PpROP-GEF4::GFP#1* cells imaged displayed essentially identical PpROP-GEF4::GFP distribution patterns (*n* = 20). Overlay (lower panels): the fluorescence images shown in the upper panels overlaid onto transmitted light reference images (differential interference contrast, DIC). Scale bars = 25 μm. **(C)** *PpROP-GEF4* expression levels in WT and “knock-in” (*PpROP-GEF4::GFP#1* and *2*) protonemata as determined by qPCR. Relative expression levels are indicated, which represent absolute *PpROP-GEF4* transcript levels normalized relative to the absolute transcript level of the reference gene *UbC-E2*. The statistical significance of differences between WT and other data sets was assessed using ANOVA (Bonferroni-Holm, one factor). ns: not significantly different. Error bars: standard deviation.

**FIGURE 4:**
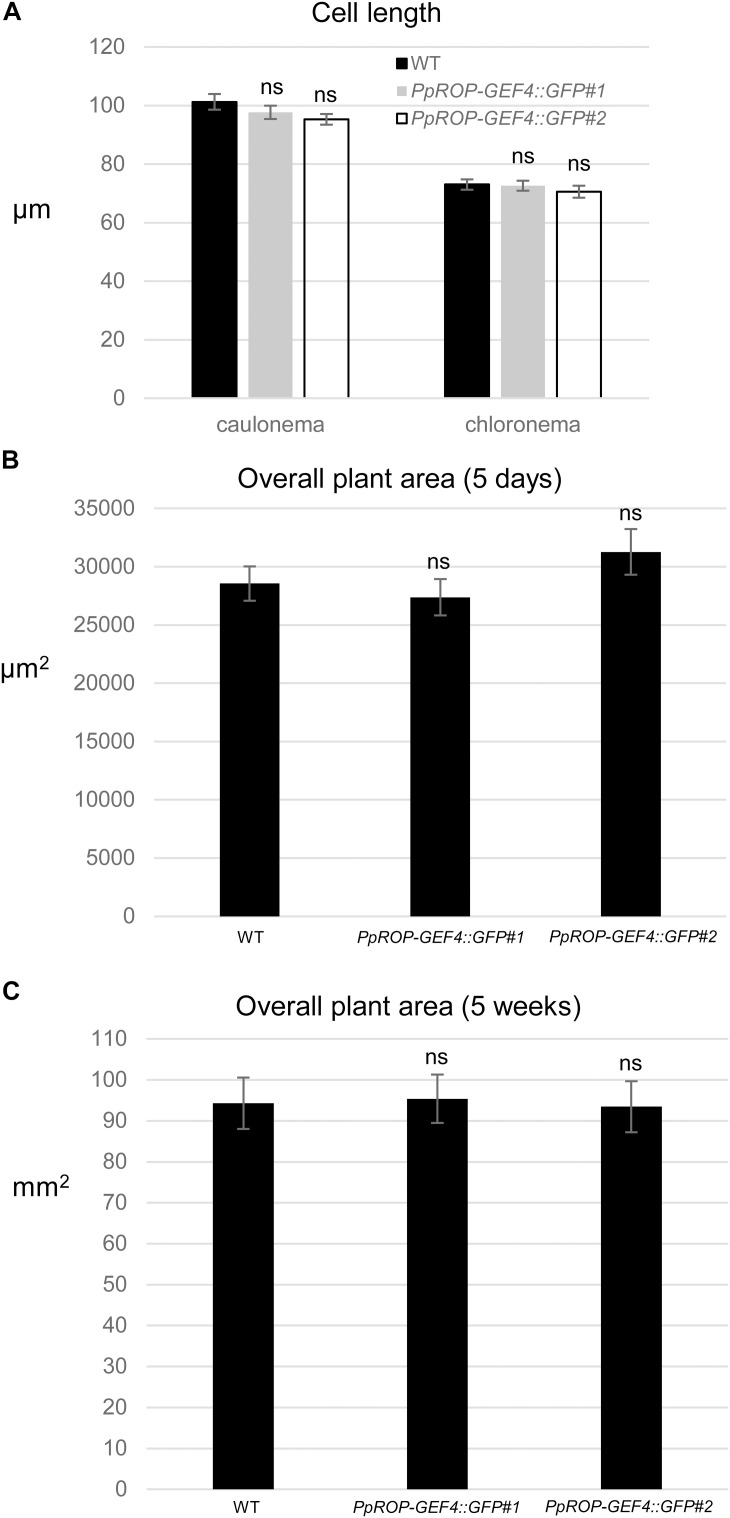
Quantitative comparison of the growth of WT and *PpROP-GEF4::GFP* “knock-in” plants. **(A)** Length of fully expanded first subapical cells of 5-day-old protonemata. **(B)** Total area of 5-day-old protonemata. **(C)** Total area of 5-week-old plants. The statistical significance of differences between WT and other data sets was assessed using ANOVA (Bonferroni-Holm, one factor). ns: not significantly different. Error bars: standard error of the mean.

**FIGURE 5:**
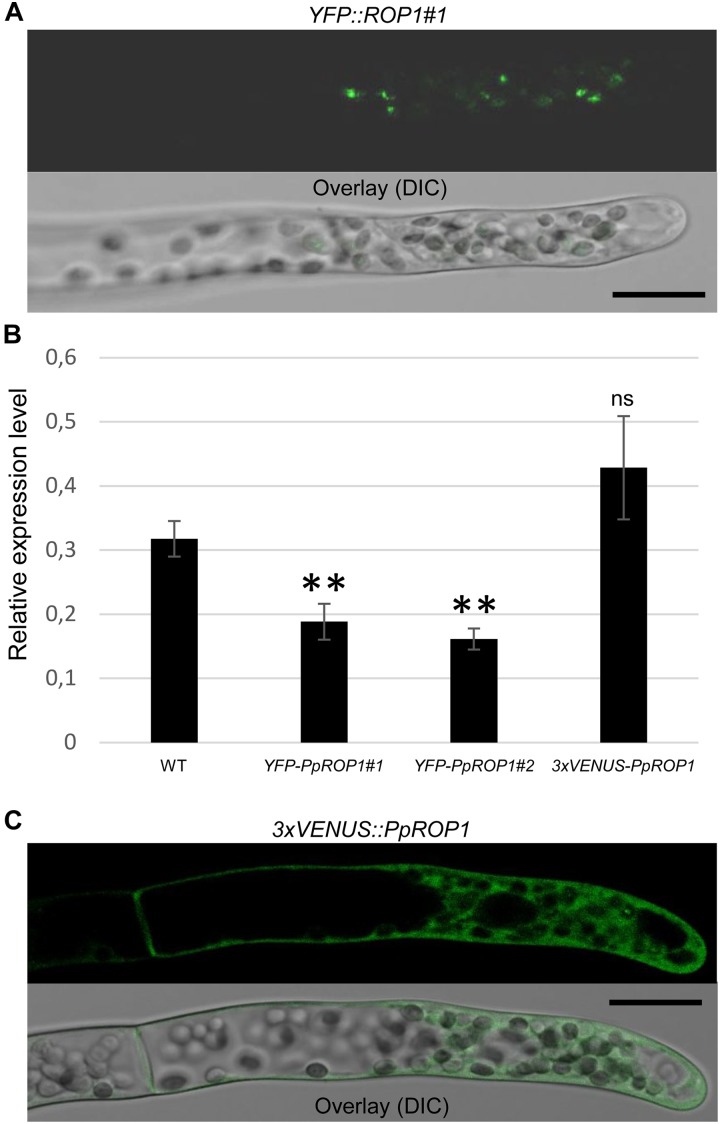
Analysis of the intracellular distribution of fluorescent PpROP1 fusion proteins in apical cells of *YFP::PpROP1* and *3xVENUS::PpROP1* “knock-in” protonemata. **(A)** Medial confocal optical section through the tip of a *YFP::PpROP1#1* protonemal filament (upper panel). All imaged apical cells did not display detectable YFP fluorescence, but similar to WT cells (Figure 3A) emitted weak background chlorophyll autofluorescence (*n* = 10). Overlay (lower panel): the fluorescence image shown in the upper panel overlaid onto a transmitted light reference image (differential interference contrast, DIC). Scale bar = 25 μm. **(B)** *PpROP1* expression levels in WT and “knock-in” (*YFP::PpROP1#1* and *2*; *3xVENUS::PpROP1*) protonemata as determined by qPCR. Relative expression levels are indicated, which represent absolute *PpROP1* transcript levels normalized relative to the absolute transcript level of the reference gene *UbC-E2*. The statistical significance of differences between WT and other data sets was assessed using ANOVA (Bonferroni-Holm, one factor). ^∗∗^*P* < 0.01; ns: not significantly different. Error bars: standard deviation. **(C)** Medial confocal optical section through the tip of a *3xVENUS::PpROP1* protonemal filament (upper panel). Only diffuse cytoplasmic 3xVENUS::PpROP1 fluorescence is visible. All imaged apical cells displayed essentially identical 3xVENUS::PpROP1 distribution patterns (*n* = 10). Overlay (lower panel): the fluorescence image shown in the upper panel overlaid onto a transmitted light reference image (differential interference contrast, DIC). Scale bar = 25 μm.

Total RNA was purified using the NucleoSpin RNA kit (Macherey-Nagel, Düren, Germany) from 7-day-old protonemata, or from leafy shoots removed without rhizoids from 5-week-old plants, which were grown as described above in the Section “Tissue Culture.” Purified RNA was incubated with 0.8 units TURBO DNase (Thermo Fisher Scientific, Waltham, MA, United States) for 15 min at room temperature to remove residual DNA. Subsequently, 1 μg purified DNA-free RNA was reverse transcribed using the iScript Reverse Transcription Supermix for RT-qPCR (Bio-Rad, Hercules, CA, United States), before the reaction mix was diluted 20 times in nuclease free water. RT-qPCR was then executed as described by Le Bail et al. (2013). Absolute quantification of transcript levels based on gDNA standard curves was performed to avoid potential effects of unequal amplification efficiencies on relative gene expression levels determined. Furthermore, 3′ UTR fragments of all analyzed transcripts were amplified to minimize potential differences in reverse transcription rates. Three identically treated replicas of each RNA sample were analyzed to account for biological variation, and each replica was run twice on the thermocycler to correct for technical variation. Obtained data were normalized based on the reference genes *PpACTIN5* (Figure 1) or *PpUbC-E2* (ubiquitin conjugation enzyme) (Figure 2–5). All primers used for qPCR analyses are listed in Supplementary Table S1.

### Expression Analysis Based on RNA Sequencing

Protonemata used for gene expression analysis based on RNA sequencing were grown from ground tissue for 7 days on solid BCD medium under long day (16 h light, 8 h darkness) conditions (Perroud et al., 2018). Average RPKM (reads per kilobase per million mapped reads) values were determined for all current *P. patens* gene models [Gene models v3.3; (Lang et al., 2018)] based on the protonemal transcriptome dataset XVIII (Perroud et al., 2018). A list of all gene models ranked according to the determined RPKM values was then established to assess the relative expression levels of *PpROP-GEF4* and *PpROP1* in comparison to the complete data set.

### Immunoblotting

*PpROP-GEF4::GFP, YFP::PpROP1*, and *YFP::PpROP1^ind^* protonemata grown for 7 days as described above in the Section “Tissue Culture” were used to analyze the fluorescent fusion proteins expressed in these protonemata based on immunoblotting. To prepare extracts from these protonemata, 200 mg (fresh weight) tissue was frozen in nitrogen and ground using a mortar and a pestle.

Ground *PpROP-GEF4::GFP* and *YFP::PpROP1* tissue was resuspended in 0.2 ml ice cold RIPA buffer (10 mM Tris pH 7.5, 150 mM NaCl, 0.5 mM EDTA, 1% triton X-100, 1% deoxycholate), which was supplemented with a protease inhibitor cocktail (“Complete,” Sigma-Aldrich, St. Louis, MO, United States) as well as with 1 mM PMSF. Resulting cell extracts were incubated at 4°C for 30 min and occasionally homogenized by up-and-down pipetting during this incubation period. Subsequently, extracts were diluted with 0.3 ml detergent-free buffer (10 mM Tris pH 7.5, 150 mM NaCl, 0.5 mM EDTA) and subjected to centrifugation at 16,000 *g* for 45 min at 4°C to collect the soluble fraction in the supernatant.

By contrast, ground *YFP::PpROP1^ind^* tissue was resuspended in IPP buffer (10 mM Tris pH 7.3, 100 mM NaCl, and 0.1% Nonidet P-40) supplemented with the same protease inhibitor cocktail (“Complete”) as indicated in the previous paragraph and immediately centrifuged at 16,000 *g* for 15 min at 4°C to collect the soluble fraction in the supernatant.

Ten microliters of prepared soluble fractions were diluted with 3× SDS Laemmli buffer, boiled at 100°C for 5 min and subject to protein separation within SDS containing 10% polyacrylamide gels. Separated proteins were either directly stained using Coomassie blue or transferred to a PVDF membrane (GE Healthcare, Munich, Germany) by semidry blotting. To detect fluorescent fusion proteins on these membranes, a rabbit anti-GFP primary antibody (10 times diluted; Sigma-Aldrich, St. Louis, MO, United States) and a HRP (horse radish peroxidase)-conjugated anti-rabbit secondary IgG antibody (1000 times diluted; Promega, Mannheim, Germany) were employed. HRP activity was visualized by incubation with luminol substrate (100 mM Tris pH 8.5, 1.25 mM luminol, 200 μM cumaric acid, 0.03% H_2_O_2_) and digital chemiluminescence imaging (Fusion SL system; Analis, Namur, Belgium).

### Yeast Two-Hybrid Assay

A cDNA fragment containing the catalytically active PRONE domain of PpROP-GEF4 (amino acids 1–453) was PCR amplified using the primers SLU445 and FAU532 and cloned between the unique *Nde*I and *Bam*HI sites of pGADT7 AD (Clontech, Mountain View, CA, United States) to generate a yeast two-hybrid prey construct. A corresponding bait construct was obtained by PCR amplifying a cDNA coding for mutant PpROP1^C194S^ specifically disrupted in posttranslational prenylation using the primers FAU524 and FAU583 and cloning it into the unique *Nde*I and *Eco*RI sites of pGBKT7 (Clontech, Mountain View, CA, United States). The sequences of all PCR primers used to generate these two constructs are listed in Supplementary Table S1. All PCR amplified fragments and junctions between ligated fragments were confirmed by Sanger sequencing.

Yeast PJ69-2A cells co-transformed with the prey and bait constructs described in the previous paragraph were plated on solid synthetic drop-out media lacking tryptophan and leucine (SD-TL), and subsequently incubated at 30°C for 3 days. Individual growing colonies were then picked, transferred to liquid SD-TL and grown overnight on a shaker (200RPM) at 30°C. Ten microliters droplets of the obtained suspension cultures were placed on solid SD-TL as well as on SD-TLAH (SD lacking tryptophan, leucine, adenine, and histidine) medium. After 1 day of incubation at 30°C, the growth of co-transformed cells was imaged using a digital camera.

### Callose Staining

Protonemata were incubated for 1 h in phosphate buffer (0.07 M Na_2_HPO_4_ pH 9) containing 0.05% aniline blue (Sigma-Aldrich, St. Louis, MO, United States) and washed three times with water before observation by confocal microscopy.

### Confocal Microscopy

Confocal imaging was performed using a TCS SP5 (Leica, Wetzlar, Germany) laser scanning microscope equipped with an HC PL APO 20 × /0.7 water immersion lens. YFP and GFP fluorescence was excited at 514 nm and imaged using a 530–600 nm emission filter. By contrast, aniline blue fluorescence was excited at 405 nm, and imaged using a 500–520 nm emission filter. Differential interference contrast (DIC) transmitted light reference images were simultaneously recorded together with all fluorescence images. TCS SP5 system software was employed to overlay DIC and fluorescence images. For display in the presented figures, the brightness of raw images was linearly adjusted using Photoshop (Adobe; San Jose, CA, United States) software.

### Measurement of the Length of Subapical Protonemal Cells and of Overall Plant Area

High-magnification bright-field transmitted light images and low-magnification chlorophyll autofluorescence images (excitation: 450–490 nm, emission: ≥515 nm) of 5-day-old plants grown as described above in the Section “Tissue Culture” were acquired using an inverted epifluorescence microscope (DMI4000B; Leica), a digital b/w camera (DFC365 FX; Leica) and an HCX PL APO CS 63x/1.20 water immersion lens, or a N PLAN 10x/0.25 lens, respectively. The ImageJ software package [1.50I; (Schneider et al., 2012)] was employed to measure the length of the first subapical cell based on high-magnification bright-field transmitted light images (*n* = 30 per cell type and genotype/condition). By contrast, low-magnification chlorophyll autofluorescence images were used to determine the overall area of imaged plants (*n* = 100 per genotype/condition) with the help of an ImageJ macro developed by Bibeau and Vidali (2014).

To determine the overall area of 5-week-old plants grown as described above in the Section “Tissue Culture,” bright-field images of these plants were recorded using a stereo microscope (M205 FA; Leica) and a digital color camera (DFC310FX; Leica). The overall area of imaged plants (*n* = 90 per genotype/condition) was measured using the ImageJ software package. Obtained images were first converted to gray scale. Thresholding was then applied to generate binary images with gray levels “0” and “1” assigned to background regions and plant area, respectively, which enabled exact measurement of plant area.

### Accession Numbers

The accession numbers of the *P. patens* genes discussed in this study are as follows: *PpROP1*: Pp3c14_4310V3.1; *PpROP-GEF1*: Pp3c2_4460V3.1; *PpROP-GEF2*: Pp3c10_9910V3.1; *PpROP-GEF3*: Pp3c1_20V3.1; *PpROP-GEF4*: Pp3c2_28420V3.1; *PpROP-GEF5*: Pp3c14_22480V3.1; *PpROP-GEF6*: Pp3c1 _36410V3.1; *PpACTIN5*: Pp3c10_17070V3.1; and *PpUbC-E2*: Pp3c14_21480V3.1.

## Results

### PpROP1 and PpROP-GEF4 Are Co-expressed in Protonemata and Interact With Each Other in Yeast Two-Hybrid Assays

Quantitative global transcriptome analysis based on RNA sequencing (RNA-Seq) indicates that all four *PpROP* genes present in the *P. patens* genome are expressed in protonemata at similar levels (*PpROP1*: 33 RPKM, *PpROP2*: 27 RPKM, *PpROP3*: 42 RPKM, and *PpROP4*: 45 RPKM [reads per kilobase per million mapped reads]; Perroud et al., 2018). As the proteins encoded by these *PpROP* genes share amino acid identities of 99–100% (Eklund et al., 2010), they are likely to display largely overlapping functions and intracellular distribution patterns. *PpROP1* and *PpROP4* code for exactly the same protein (100% amino acid identity), which based on the RNA-Seq data discussed above represents the most abundant PpROP isoform expressed in protonemata. In this study, we choose to analyze the intracellular localization of PpROP1 as a representative of the PpROP protein family.

The *P. patens* genome contains six homologous proteins displaying high similarity to ROP-GEFs (PpROP-GEF1-6) (Eklund et al., 2010), which appear likely to non-specifically target all PpROPs given the extraordinarily high amino acid identities shared by these proteins. In order to assess the potential importance of each of these PpROP-GEFs in the control of PpROP activity during tip growth, quantitative PCR (qPCR) was employed to compare transcript levels in protonemata, which elongate based on tip growth of apical cells, and in leafy shoots without rhizoids, which do not contain tip-growing cells. Although expression of all six *PpROP-GEF* genes was detected in both analyzed tissues, *PpROP-GEF3-6* displayed substantially higher transcript levels in protonemata as compared to *PpROP-GEF1* and *2* (Figure 1A). Furthermore, only *PpROP-GEF4* was found to be significantly more strongly expressed in protonemata than in leafy shoots (Figure 1A). These observations are consistent with previously published RNA-Seq data (Perroud et al., 2018) and suggest that PpROP-GEF4 in particular may have important functions in the control of PpROP activity in tip-growing cells. To support this interpretation, the catalytically active PRONE domain (Plant-specific Rop Nucleotide Exchanger) (Gu et al., 2006) of PpROP-GEF4 was demonstrated to interact with PpROP1 in yeast two-hybrid assays (Figure 1B).

### With Regards to Transcript Level, *PpROP1* and *PpROP-GEF4* Rank Among to Top 20 or 60%, Respectively, of All Genes Active in Protonemata

qPCR analysis showed that *PpROP1* transcripts are about twice as abundant as *PpROP-GEF4* transcripts in protonemata (Figure 2). Consistent with somewhat higher *PpROP1* transcript levels, quantitative global transcriptome analysis based on RNA sequencing (RNA-Seq; Perroud et al., 2018), which was performed using protonemata grown under similar conditions as employed to generate the qPCR data, indicated that *PpROP1* is roughly 1.8× more strongly expressed in protonemata as compared to *PpROP-GEF4* (33 and 18 RPKM, respectively; Perroud et al., 2018). Interestingly, the RNA-Seq analysis further suggested that with regards to transcript level *PpROP1* and *PpROP-GEF4* rank among the top 20 or 60%, respectively, of all 23612 genes active in protonemal cells. This indicates relatively high expression of *PpROP1* and moderate expression of *PpROP-GEF4* in these cells.

### Analysis of the Intracellular Localization of PpROP1 and PpROP-GEF4 Based on YFP or GFP “Knock-In”

A “knock-in” strategy was pursued to generate two independent *P. patens* lines (*PpROP-GEF4::GFP#1* and *#2*) expressing PpROP-GEF4 with GFP [“soluble-modified red-shifted” smRS-GFP; (Davis and Vierstra, 1998)] attached at the C-terminus (PpROP-GEF4::GFP) under the control of the native genomic environment of the *PpROP-GEF4* gene. PCR based genotyping confirmed correct genomic integration of the GFP cDNA in both these lines (Supplementary Figure S1). Confocal imaging established that by contrast to WT protonemata, which only emitted weak background chlorophyll autofluorescence (Figure 3A), in both *PpROP-GEF4::GFP* lines PpROP-GEF4::GFP was detectably expressed exclusively in apical cells of protonemata, and specifically accumulated at the plasma membrane of these cells within a small dome-shaped domain at the extreme tip (Figure 3B). PpROP-GEF4::GFP localization in cells displaying choloronemal and caulonemal characteristics was indistinguishable from each other. *PpROP-GEF4* transcript levels in WT and *PpROP-GEF4::GFP#1* or *#2* protonemata were not significantly different as determined by qPCR (Figure 3C). Furthermore, a quantitative morphological analysis of WT and *PpROP-GEF4::GFP#1* and *#2* plants demonstrated that GFP “knock-in” into the *PpROP-GEF4* gene neither affected the length of the fully expanded first sub-apical cell in 5-day-old protonemata displaying either chloronemal or caulonemal characteristics, nor the overall size of 5-day or 5-week-old moss plants (Figure 4).

**FIGURE 6:**
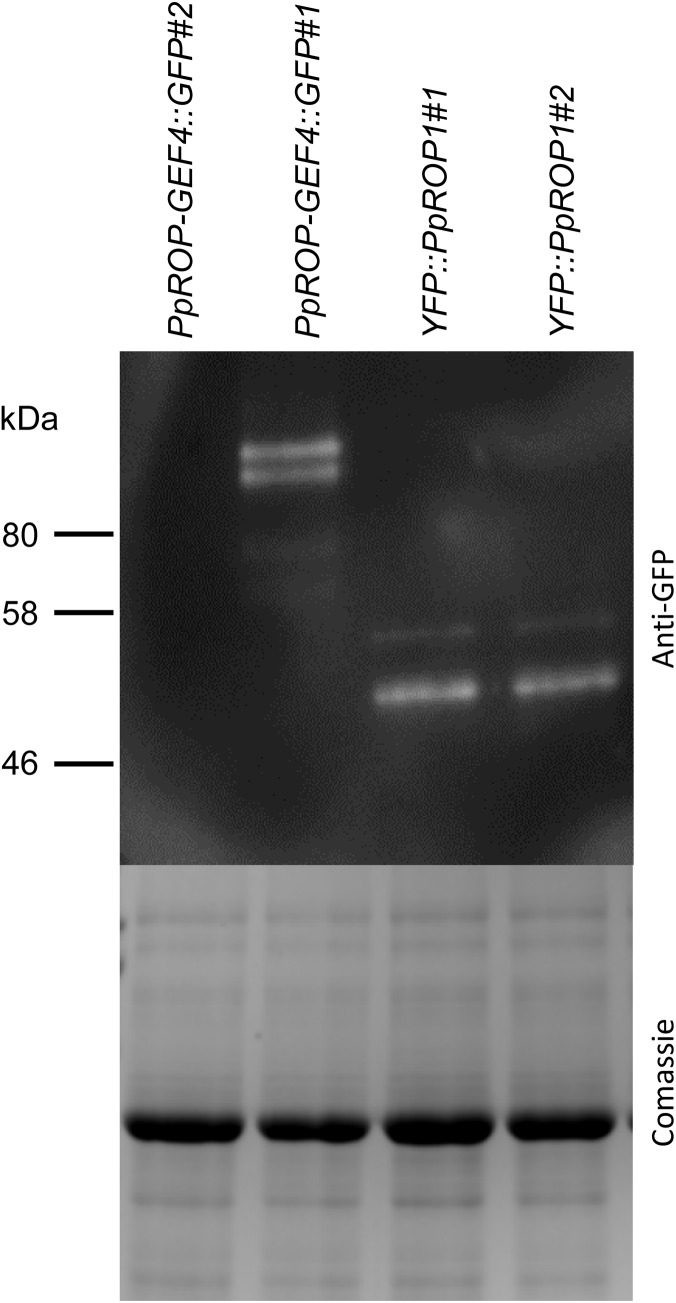
PpROP-GEF4::GFP and YFP::PpROP1 protein levels in “knock-in” protonemata. Total proteins extracted from *PpROP-GEF4::GFP* and *YFP::PpROP1* protonemata were separated by SDS-PAGE and either analyzed by immunoblotting using an anti-GFP antibody (upper panel; chemiluminescence detection), or coomassie stained to show equal loading of all lanes (lower panel). Predicted sizes of PpROP-GEF4::GFP and YFP::PpROP1 are 91 and 50 kDa, respectively.

In parallel, “knock-in” lines expressing YFP (“enhanced” eYFP; BD Biosciences-Clontech; San Jose, CA, United States) fused to PpROP1 were produced. Posttranslational modification of the C-terminal CAAX box of ROP GTPases, which encompasses cysteine prenylation and proteolytic removal of the last three amino acid residues (AAX), is essential for the membrane association of these proteins (Sorek et al., 2011). Therefore, YFP needed to be attached to the N-terminus of PpROP1. To this end, we employed simultaneous co-transformation with two separate constructs, one designed to “knock-in” a YFP cDNA at the 5′ end of the first exon of the PpROP1 gene, and the other to insert a selectable marker gene into the Pp108B intergenic region of the *P. patens* genome (Schaefer and Zrÿd, 1997). Using this strategy, two independent “knock-in” lines expressing YFP::PpROP1 under the control of the native genomic environment of the *PpROP1* gene were generated (*YFP::PpROP1#1* and *#2*), which both carried a correctly inserted copy of the YFP cDNA as determined by PCR based genotyping (Supplementary Figure S2). Unfortunately, YFP fluorescence above background chloroplast autofluorescence also visible in WT cells (Figure 3A) was not detectable by confocal microscopy in *YFP::PpROP1#1* or *#2* protonemata (Figure 5A). qPCR analysis showed that *YFP::PpROP1* transcript levels in both “knock-in” lines were slightly, but statistically significantly reduced as compared to WT *PpROP1* levels (Figure 5B). Counting in the somewhat higher transcript levels of *PpROP1* as compared to *PpROP-GEF4* in WT protonemata (Figure 2), based on qPCR analysis, YFP::PpROP1 (Figure 5B), and PpROP-GEF4::GFP (Figure 3) appear to be expressed at similar levels in the analyzed “knock-in” lines (*YFP::PpROP1* and *PpROP-GEF4::GFP*, respectively). Immunoblotting with a GFP antibody confirmed that this was in fact the case (Figure 6), although PpROP-GEF4::GFP was only detectable in *PpROP-GEF4::GFP#1* protonemata using this technique. For unclear reasons, *PpROP-GEF4::GFP#2* protonemata displayed patchy PpROP-GEF4::GFP expression only detectable by confocal microscopy in some filaments, and consequently failed to produce sufficient amounts of this fusion protein to enable immunoblot detection. Immunoblotting further established that in the analyzed “knock-in” protonemata both PpROP-GEF4::GFP and YFP::PpROP1 predominantly accumulated as intact, full-length fusion proteins with predicted sizes of roughly 91 and 50 kDa, respectively. Interestingly, PpROP-GEF4::GFP was consistently detected as a double band indicating that this protein may exist in two forms possibly due to alternative use of different translational START codons, proteolytic processing or posttranslational modification.

As discussed in the previous paragraph, YFP::PpROP1 and PpROP-GEF4::GFP were expressed at similar levels in the analyzed “knock-in” protonemata. Furthermore, YFP and GFP are both brightly fluorescent and essentially equally readily detectable by confocal microscopy (Rizzo et al., 2004; Day and Davidson, 2009). Based on these considerations, it appears likely that PpROP-GEF4::GFP was detectable by confocal microscopy in “knock-in” protonemata because it strongly accumulated at the plasma membrane within a small apical domain, whereas YFP::PpROP1 displayed a wider distribution pattern and therefore failed to reach local concentrations above the detection limit of this technique. In fact, a large percentage of the total amount of ROP GTPases in plant cells has been shown to be present in the cytoplasm Kost et al. (1999) presumably in the form of soluble heterodimers with ROP-GDI proteins.

Based on essentially the same “knock-in” strategy as employed to generate *YFP::PpROP1* lines, a *3xVENUS::PpROP1* line was developed, which carried a correctly inserted *3xVENUS* cDNA (Supplementary Figure S2) and expressed essentially at endogenous levels (Figure 5B) PpROP1 attached at the N-terminus to three fused copies of VENUS, a YFP variant undergoing fast and efficient maturation (Nagai et al., 2002). Although based on confocal microscopy diffuse cytoplasmic accumulation of 3xVENUS::PpROP1 was clearly visible in apical cells of “knock-in” protonemata, accumulation of this protein at the plasma membrane was not detected (Figure 5C). As overexpressed PpROP fused to a single copy of cerulean (Ito et al., 2014) or YFP (see below) effectively accumulates at the plasma membrane of *P. patens* protonemata, the observed 3xVENUS::PpROP1 distribution pattern suggests that the large 3xVENUS tag interferes with the membrane association of this fusion protein.

### Analysis of the Intracellular Localization of PpROP1 Based on Estradiol-Titratable Expression of a YFP Fusion Protein

**FIGURE 7:**
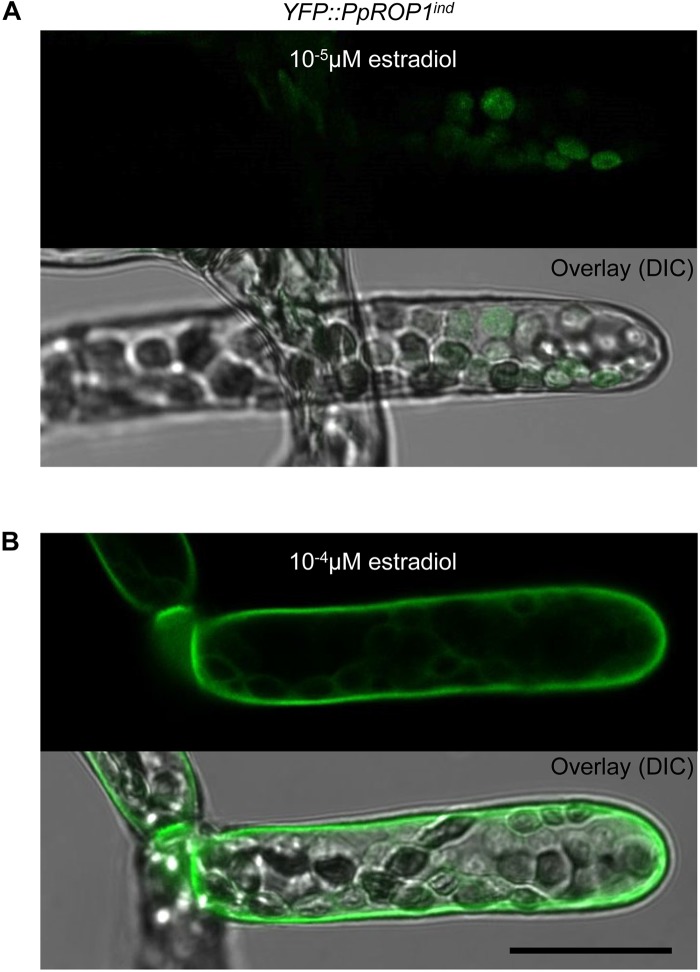
Analysis of the intracellular distribution of YFP::PpROP1 expressed at different levels in apical cells of *YFP::PpROP1^ind^* protonemata induced with 10^-5^ or 10^-4^ μM estradiol. Upper panels in **(A,B)**: Medial confocal optical section through the tip of *YFP::PpROP1^ind^* protonemal filaments grown in the presence of 10^-5^ μM **(A)** or 10^-4^ μM **(B)** estradiol. Only background chlorophyll autofluorescence also displayed by WT cells (Figure 3A) was visible after induction with 10^-5^ μM estradiol **(A)**. At each of the two estradiol concentration tested, all imaged apical cells displayed essentially identical fluorescence emission patterns (*n* = 20). Lower panels (Overlay) in **(A,B)**: fluorescence images shown in the upper panels overlaid onto transmitted light reference images (differential interference contrast, DIC). Scale bar = 25 μm.

Results presented in the previous section demonstrate that YFP::PpROP1 is not detectable by confocal imaging when expressed at essentially endogenous levels in *P. patens* “knock-in” protonemata. We therefore decided to employ estradiol-titratable YFP::PpROP1 expression to investigate the intracellular distribution of this fusion protein. Conceptually, this approach enables imaging of YFP::PpROP1 at the lowest detectable expression level, which minimizes potential overexpression effects on the structural organization of observed cells and on YFP:PpROP1 distribution within these cells. To this end, an estradiol-activated chimeric transcription factor (XVE) was constitutively expressed in *P. patens* protonemata along with YFP::PpROP1 under the control of an artificial, XVE-responsive promoter (Zuo et al., 2000; Kubo et al., 2013). A transgenic *P. patens* line (*YFP::PpROP1^ind^*) was generated, which carried within the neutral PIG1 genomic region a single copy of a construct containing a constitutively active XVE expression cassette (GX8 promoter) along with the XVE-responsive, estradiol inducible promoter fused to the 5′ end of a *YFP::PpROP* cDNA (Supplementary Figure S3). *YFP:PpROP1^ind^* protonemata grown for 5 days in the presence of estradiol at a concentration of 10^-5^ μM, 10^-4^ μM or higher were analyzed by confocal imaging. Whereas 10^-5^ μM estradiol did not detectably induce YFP:PpROP1 expression (Figure 7A), in protonemata treated with 10^-4^ μM estradiol this fusion protein was observed to evenly accumulate at plasma membrane along the entire cell circumference not only in the apical but also in all other cells (Figure 7B). Higher estradiol concentrations enhanced the brightness of YFP::PpROP1 fluorescence emission, but did not change the observed distribution pattern of this fusion protein (Supplementary Figure S4).

**FIGURE 8:**
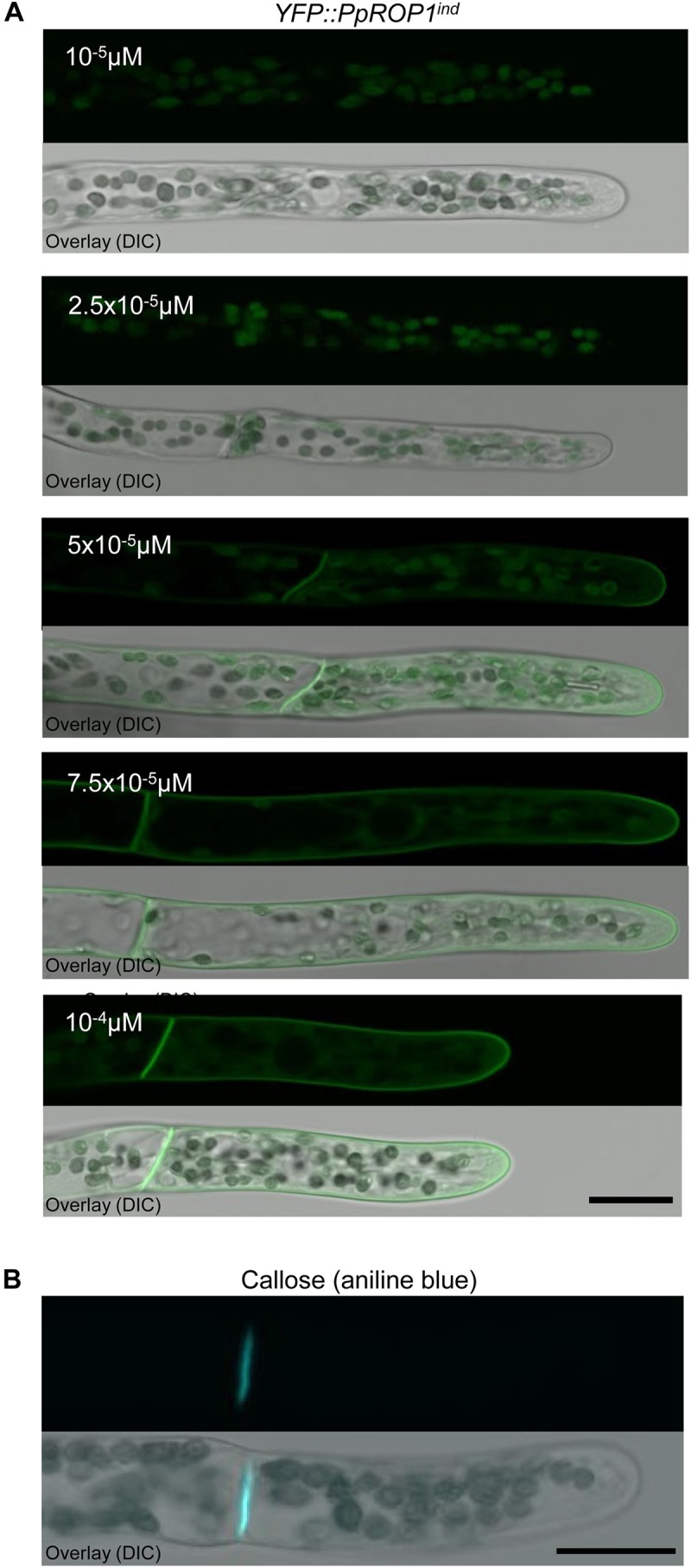
Analysis of callose accumulation in the cell wall and of the intracellular distribution of YFP::PpROP1 expressed at different levels in apical cells of *YFP::PpROP1^ind^* protonemata induced with estradiol at a range of concentrations. **(A)** Medial confocal optical sections through the tips of *YFP::PpROP1^ind^* protonemal filaments grown in the presence of estradiol at the indicated concentrations (upper panels). Only background chlorophyll autofluorescence also displayed by WT cells (Figure 3A) was visible after induction with 10^-5^ M or 2.5 × 10^-5^ M estradiol. At each of the estradiol concentration tested, all imaged apical cells displayed essentially identical fluorescence emission patterns (*n* = 20). Overlay (lower panel): the fluorescence image shown in the upper panel overlaid onto a transmitted light reference image (differential interference contrast, DIC). Scale bar = 25 μm. **(B)** Medial confocal optical section through the tip of a WT protonemal filament stained with aniline blue to visualize callose in the cell wall. All imaged apical cells displayed essentially identical anline blue staining patterns (*n* = 14). Overlay (lower panel): the fluorescence image shown in the upper panel overlaid onto a transmitted light reference image (differential interference contrast, DIC). Scale bar = 25 μm.

To identify the lowest detectable level of YFP::PpROP1 expression, *YFP::PpROP1^ind^* protonemata were treated for 5 days with 10^-5^, 2.5 × 10^-5^, 5 × 10^-5^, 7.5 × 10^-5^, or 10^-4^ μM estradiol before confocal imaging. The lowest estradiol concentration that detectably induced YFP::PpROP1 expression was found to be 5 × 10^-5^ μM (Figure 8A). At this concentration, plasma membrane association of YFP::PpROP1 was specifically observed in a dome-shaped region at the tip of apical cells, as well as along the cross walls that are separating these cells from their directly adjacent neighbors within protonemal filaments (Figure 8A). These cross walls are derived from phragmoplast-mediated cell plate construction during cytokinesis and are strongly enriched in callose (Figure 8B). YFP::PpROP1 localization was indistinguishable in cells displaying chloronemal or caulonemal characteristics. Interestingly, under the described conditions YFP::PpROP1 associated with a substantially more extended region of the apical plasma membrane region as compared to PpROP-GEF4::GFP (compare Figure 8A, 3A). At 7.5 × 10^-5^ μM estradiol, YFP::PpROP1 labeled the entire plasma membrane of all protonemal cells, although enhanced plasma membrane association of this fusion protein at the tip of apical cells was still discernible (Figure 8A). By contrast, as described above (Figure 7B), after treatment with 10^-4^ μM estradiol even labeling of the entire plasma membrane of all protonemal cells was observed (Figure 8A).

**FIGURE 9:**
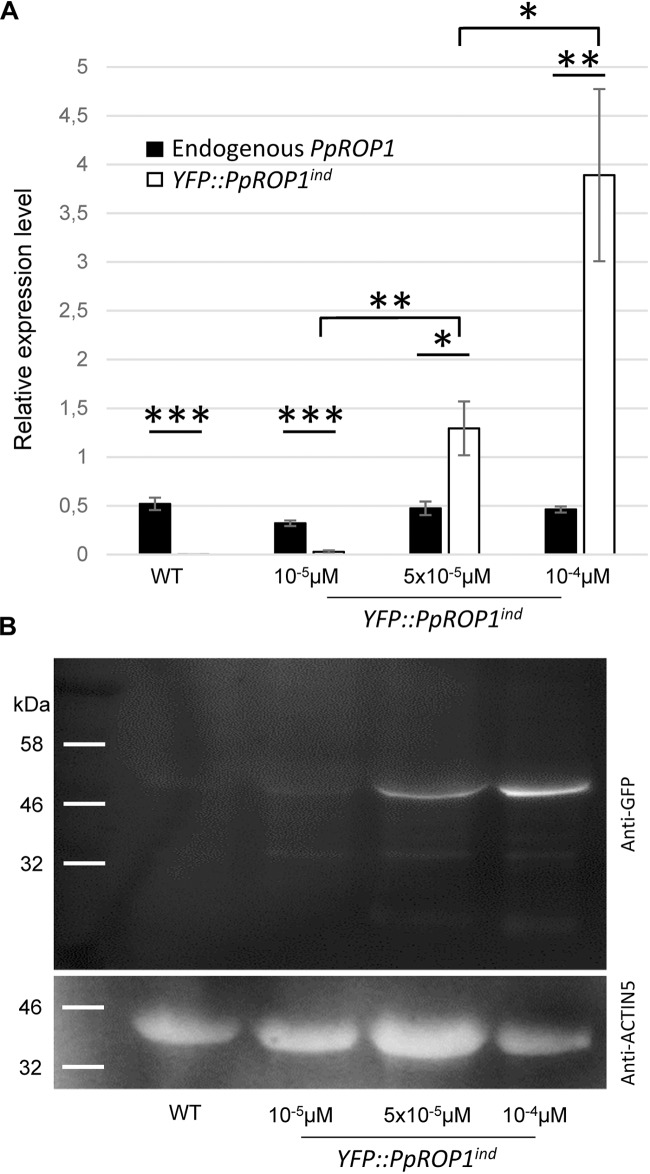
*PpROP1* and *YFP::PpROP1* transcript levels, as well as YFP::PpROP1 protein levels, in *YFP::PpROP1^ind^* protonemata induced with 10^-5^ μM, 5 × 10^-5^ μM, or 10^-4^ μM estradiol. **(A)** Endogenous *PpROP1* and inducible *YFP::PpROP1* expression levels as determined by qPCR in WT protonemata and in *YFP::PpROP1^ind^* protonemata grown in the presence of estradiol at the indicated concentrations. Relative expression levels are indicated, which represent absolute *PpROP1* and *YFP::PpROP1* transcript levels normalized relative to the absolute transcript level of the reference gene *UbC-E2*. The statistical significance of differences between all data sets was assessed using ANOVA (Bonferroni-Holm, one factor). ^∗^*P* < 0.05; ^∗∗^*P* < 0.01; ^∗∗∗^*P* < 0.001: statistically significant differences relevant in the context of this study. Error bars: standard deviation. **(B)** Total proteins extracted from WT protonemata and from *YFP::PpROP1^ind^* protonemata grown in the presence of estradiol at the indicated concentrations were separated by SDS-PAGE and analyzed by immunoblotting using either an anti-GFP (upper panel) or an anti-actin (lower panel) antibody (chemiluminescence detection). Immunoblotting using the anti-actin antibody served as loading control. Predicted sizes of YFP::PpROP1 and PpACTIN5 are 50 and 42 kDa, respectively.

qPCR analysis showed that the expression of the endogenous *PpROP1* gene remained unaffected in *YFP::PpROP1^ind^* protonemata treated with 10^-5^, 5 × 10^-5^, or 10^-4^ μM estradiol (Figure 9A). Furthermore, consistent with the imaging data shown in Figure 7, 8, no substantial induction of *YFP::PpROP1* transcript levels was detected in the presence of 10^-5^ μM estradiol, whereas after treatment with 5x10^-5^ or 10^-4^ μM estradiol *YFP::PpROP1* expression was massively enhanced and reached roughly 3× or 8× higher levels, respectively, as compared to endogenous *PpROP1* expression. Investigation of protein extracts prepared from estradiol treated *YFP::PpROP1^ind^* protonemata using immunoblotting and a GFP antibody (Figure 9B) confirmed that YFP::PpROP1 protein levels were in fact induced as indicated by qPCR based analysis of transcript levels (Figure 9A). This investigation further established that YFP::PpROP1 predominantly accumulated within estradiol treated protonemata as an intact, full-length fusion protein with the predicted size of 50 kDa.

**FIGURE 10:**
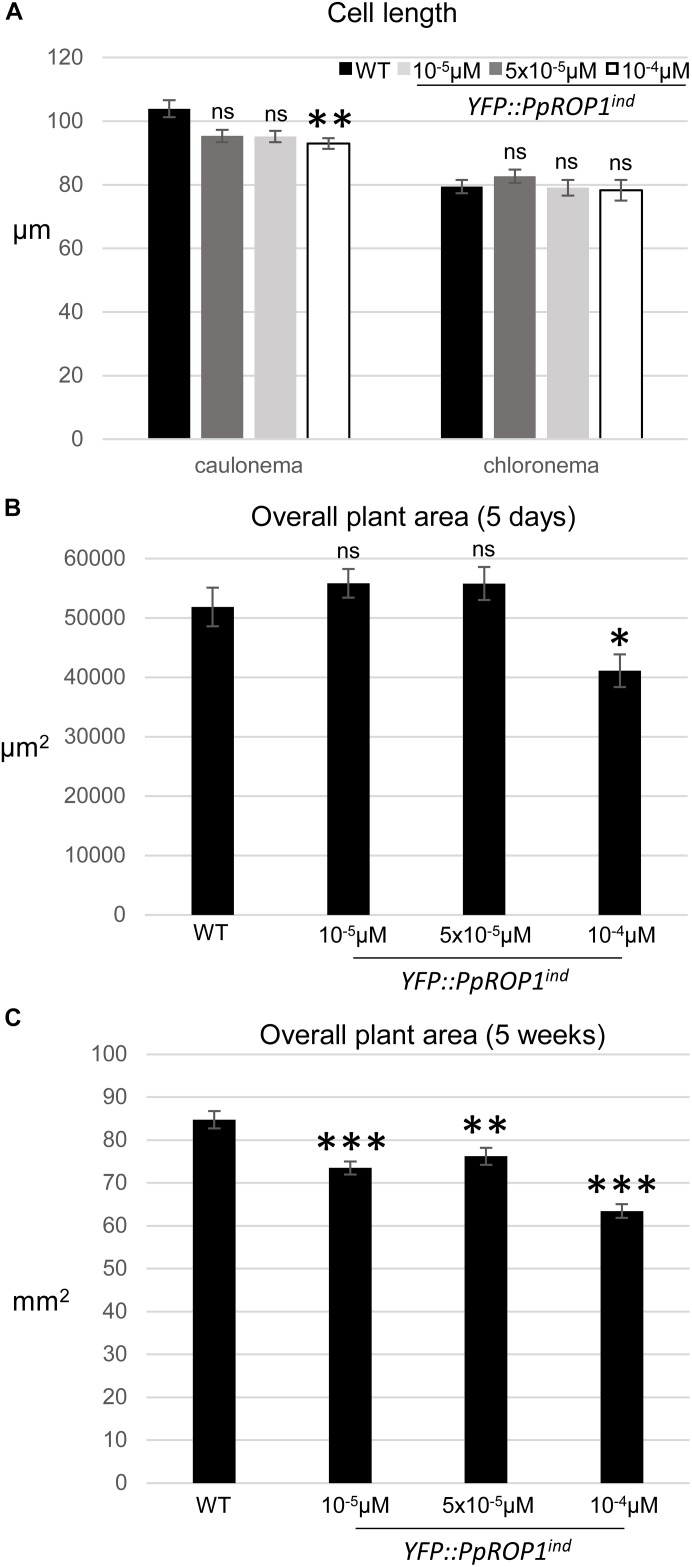
Quantitative comparison of the growth of WT plants and of *YFP::PpROP1^ind^* plants induced with 10^-5^ μM, 5 × 10^-5^ μM, or 10^-4^ μM estradiol. **(A)** Length of fully expanded first subapical cells of 5-day-old protonemata. **(B)** Total area of 5-day-old protonemata. **(C)** Total area of 5-week-old plants. The statistical significance of differences between WT and other data sets was assessed using ANOVA (Bonferroni-Holm, one factor). ^∗^*P* < 0.05; ^∗∗^*P* < 0.01; ^∗∗∗^*P* < 0.001; ns: not significantly different. Error bars: standard error of the mean.

In summary, data presented above demonstrate that based on confocal imaging of *P. patens* protonemata YFP::PpROP1 (a) is not detectable at essentially endogenous expression levels after YFP “knock-in” (Figure 5), (b) can readily be observed when expressed at roughly three times higher levels under the control of an estradiol-inducible promoter (Figure 8A, 9), and (c) displays altered distribution patterns at even higher expression levels (Figure 7, 8A, 9 and Supplementary Figure S4). Next, we wanted to confirm that at the minimal detectable expression level (roughly 3× higher as compared to endogenous PpROP1), YFP::PpROP1 has no major effects on the development of *P. patens* protonemata and therefore is likely to display an essentially normal intracellular distribution. To this end, a quantitative morphological characterization of *YFP::PpROP1^ind^* protonemata grown for 5 days or 5 weeks in the presence of 10^-5^, 5 × 10^-5^, or 10^-4^ μM estradiol was performed. In fact, after 5 days of estradiol treatment at a concentration of 5 × 10^-5^ μM, which enabled confocal YFP::PpROP1 imaging at the minimal detectable expression level (Figure 8A), neither the length of the first sub-apical cell of protonemata with chloronemal or caulonemal characteristics, nor overall plant size, were detectably affected (Figure 10A,B). In the presence of 10^-5^ or 5 × 10^-5^ μM estradiol, a small but statistically significant reduction of overall plant size was only observed after prolonged incubation for 5 weeks (Figure 10C). By contrast, higher levels of YFP::PpROP1 expression induced by 10^-4^ μM estradiol (Figure 9) detectably reduced both the length of the first sub-apical cell of protonemata with caulonemal characteristics, as well as overall plant size, already after 5 days (Figure 10A,B) and also caused a pronounced decrease in overall plant size after 5 weeks (Figure 10C).

## Discussion

Through the development and characterization of GFP “knock-in” lines, we have established that a PpROP-GEF4::GFP fusion protein expressed at endogenous levels in normally developing *P. patens* protonemata specifically accumulates at the plasma membrane of apical cells displaying either chloronemal or caulonemal characteristic within a small dome-shaped region at the extreme apex (Figure 3). By contrast, expression of a YFP::PpROP1 fusion protein at essentially endogenous levels in *P. patens* “knock-in” protonemata was found to be below the limit of detection by confocal microscopy. To circumvent this problem, we have adapted and employed a system developed for estradiol-titratable gene expression in vascular plants, which enabled us to show that YFP::PpROP1 also accumulates in a dome-shaped plasma membrane region at the extreme tip of apical protonemal cells (Figure 8) with chloronemal or caulonemal characteristics when expressed at the minimal detectable level (roughly 3× higher as compared to endogenous expression), at which *P. patens* development was affected only marginally and after prolonged incubation. Interestingly, the apical plasma membrane domain with which YFP::PpROP1 is associated was found to be substantially larger as compared to the PpROP-GEF4::GFP labeled region. In summary, based on fluorescent protein tagging under the best achievable conditions, PpROP1 and PpROP-GEF4, two proteins that potentially play key roles in the control of tip growth, have been demonstrated to co-localize specifically at the plasma membrane at the tip of apical protonemal cells, where directional cell expansion occurs. These results confirm and substantially extend previously reported data concerning ROP and ROP-GEF intracellular distribution in tip growing cells, which are based on immunolabeling after chemical fixation that only suboptimally preserves the structure of these cells (Lin et al., 1996; Molendijk et al., 2001), or on the imaging of fluorescent fusion proteins that were ectopically expressed and/or overexpressed at undefined levels (Kost et al., 1999; Chang et al., 2013).

At the tip of growing pollen tubes, specific association of ROP GTPases with the apical plasma membrane appears to be dynamically maintained based on the following recycling mechanism (Kost, 2008): ROP inactivation by ROP-GAPs at the flanks of the tip is followed by ROP-GDI mediated ROP extraction from the plasma membrane and recycling back to the extreme apex. At this location, inactive ROP reassociates with the plasma membrane and subsequently undergoes ROP-GEF dependent reactivation. The intracellular distributions of PpROP1 and PpROP-GEF4 observed in the study presented here are perfectly consistent with this proposed recycling mechanism. Co-localization of these two proteins at the plasma membrane at the extreme apex of tip-growing apical protonemal cells indicates that PpROP1 may be activated by PpROP-GEF4 within this domain of co-localization. Furthermore, the dome-shaped apical plasma membrane domain with which PpROP1 is associated laterally extends substantially beyond the similarly shaped region of PpROP-GEF4 accumulation, suggesting that within the lateral PpROP-GEF4-free plasma membrane region inactive PpROP1 may accumulate. It will be interesting to test whether active and inactive forms of PpROP1 are in fact spatially segregated within the dome-shaped PpROP1 labeled plasma membrane region at tip of apical protonemal cells. To this end, the intracellular distributions in these cells of *P. patens* ROP-GAP homologs as well as of markers specific for active PpROP1 [fluorescent CRIB-domain fusion proteins (Hwang et al., 2005) or FRET sensors (Wong et al., 2018)] can be investigated using the tools and methods developed in the course of the study. In any case, a possible function of PpROP-GEF4 in PpROP1 activation in apical protonemal cells is further supported also by the observations that both proteins are highly and preferentially expressed in these cells as determined based on qPCR analysis and confocal imaging of “knock-in” lines, and interact with each other in yeast two-hybrid assays.

Interestingly, in apical protonemal cells PpROP1 was found to specifically accumulate at the plasma membrane not only at the growing tip, but also along the cross walls that are separating these cells from their directly adjacent subapical neighbors. We found that these cross walls, which are derived from phragmoplast-mediated cell plate formation during cytokinesis, are strongly enriched in callose (Figure 8). Furthermore, published reports have demonstrated (a) that PpROP overexpression interferes with cross wall formation in *P. patens* protonemata (Ito et al., 2014), and (b) that active forms of closely related yeast Rho (Qadota et al., 1996) and plant ROP GTPases (Hong et al., 2001) directly interact with and regulate plasma membrane associated callose synthases. Together, all these observations indicate possible functions of PpROP1 in regulation of cross walls-specific callose deposition, which may occur either during or after cytokinesis.

Although data presented here establish that GFP “knock-in” into the *PpROP-GEF4* gene, or YFP::PpROP1 expression at minimal levels detectable by confocal imaging, have no substantial effects on protonemal development, full functionality of the analyzed PpROP-GEF4::GFP or YFP::PpROP1 fusion proteins could unfortunately not be demonstrated. Neither *pprop-gef4* nor *pprop1* knock-out mutants generated in the course of this study displayed detectable phenotypes that potentially could have been complemented by PpROP-GEF4::GFP or YFP::PpROP1 expression, respectively. The generation of knock-out mutants missing multiple *PpROP-GEF* or *PpROP* genes is beyond the scope of this study. Even if such mutants were available, it may not be possible to complement potential phenotypes by the expression of single members of the PpROP-GEF or PpROP protein families. In fact, the expression of single PpROPs failed to restore normal protonemal development in *P. patens* mutants in which the entire *PpROP* gene family had been transiently silenced based on RNAi (Burkart et al., 2015).

In addition to providing important insights concerning the intracellular distributions and cellular functions of PpROP1 and PpROP-GEF4, two proteins apparently playing key roles in the control of tip growth in *P. patens* protonemata, the study presented here also established an optimal alternative approach that can be employed to investigate the intracellular localization of *P. patens* proteins, which are not expressed at sufficiently high levels for the successful application of fluorescent protein “knock-in” strategies. Our results indicate that “knocking-in” cDNAs coding for multiple fused copies of fluorescent proteins to enhance the brightness of fusion proteins has only limited potential to overcome this problem. The large fluorescent tags generated as a result of this strategy are likely to interfere with the correct intracellular targeting not only of 3xVENUS::PpROP1 as observed in this study, but also of other fusion proteins. Furthermore, we found that effective fluorescent protein tagging based on “knock-in” strategies requires endogenous expression of target proteins at relatively high levels (see also below). If endogenous expression levels are low, tags composed of multiple copies of fluorescent proteins may therefore not sufficiently enhance the brightness of fusion proteins to overcome the detection limit.

By contrast, we demonstrate that estradiol-titratable overexpression of fluorescent fusion proteins at the minimal detectable level represents a powerful alternative approach to determine the intracellular localization of *P. patens* proteins that are expressed at too low levels to be detectable after fluorescent protein “knock-in.” This alternative approach was successfully applied to establish the intracellular distribution of a YFP::PpROP1 fusion protein, which as discussed above was not detectable by confocal microscopy at the endogenous expression level, but could readily be imaged at roughly 3× higher expression levels in essentially normally developing *P. patens* protonemata. At even higher expression levels (i.e., roughly 8× the endogenous level), YFP::PpROP1 not only displayed a massively altered intracellular distribution pattern, but also substantially reduced cellular and protonemal growth. When estradiol-titratable overexpression is employed to investigate the intracellular localization of fluorescent fusion protein, it is therefore absolutely essential (a) to determine the minimal detectable expression level, and (b) to confirm that at this expression level functions and development of analyzed cells or tissue remain unaffected. Individual fluorescent fusion protein proteins may turn out to disrupt cellular functions and/or development already at the minimal detectable expression level. In such cases, alternative protein localization methods (e.g., immunolabeling) not depending on fluorescent protein tagging need to be employed. It is currently not possible to predict with certainty, if and how frequently this problem may be encountered. However, although ROP GTPases are highly active proteins generally displaying strong overexpression phenotypes (Ito et al., 2014), we demonstrate that the intracellular distribution of YFP::PpROP1 can be imaged essentially non-invasively at the minimal detectable expression level. This indicates that it should be possible to non-invasively characterize the intracellular localization of many proteins based on fluorescent protein tagging and estradiol-titratable expression. A potential disadvantage of this approach is that it exclusively enables the investigation of intracellular protein distributions, at least when applied as described in this study, whereas fluorescent protein “knock-in” in addition allows the characterization of gene expression patterns. To address this issue it may be possible to replace the constitutive promoter driving the expression of the estradiol-responsive chimeric transcription factor (XVE) by the endogenous promoter of the gene coding for the protein, whose intracellular localization is investigated.

Interestingly, YFP::ROP1 was not detectable by confocal microscopy when expressed at essentially endogenous levels in “knock-in” protonemata, although as indicated by RNA-Seq-based global transcriptome analysis *PpROP1* ranks among the top 20% of all 23’612 genes active in *P. patens* protonemata with regards to transcript level. This suggests that a substantial proportion of all *P. patens* genes may be expressed at levels too low to enable analysis of protein localization based on fluorescent protein “knock-in.” In fact, only few reports describing the successful application of this approach have been published to date (e.g., Perroud and Quatrano, 2006, 2008). However, in addition to transcript level other factors including protein stability and intracellular distribution also influence the detectability of individual fluorescent fusion proteins at endogenous expression levels. We have, for example, been able to image the intracellular distribution of a PpROP-GEF4::GFP fusion protein expressed at endogenous levels in “knock-in” protonemata, although *PpROP-GEF4* displays a somewhat lower transcript level as compared to *PpROP1*. This is presumably due to the highly specific association of PpROP-GEF4::GFP with the plasma membrane of apical protonemal cells exclusively at the extreme apex, which results in local accumulation of this fusion to detectable levels. As compared to PpROP-GEF4::GFP, YFP::ROP1 is more widely distributed within these cells and accumulates not only at a more extended region of the plasma membrane (this study), but presumably also in the cytoplasm (Kost et al., 1999; Sun et al., 2015). In any case, based on RNA-Seq analysis *PpROP-GEF4* ranks among the top 60% of all genes active in *P. patens* protonemal cells with respect to transcript level, indicating that in these cells a substantial number of genes are expressed at lower levels than *PpROP-GEF4*. Together, the results of our study suggest that determining the intracellular distribution of the proteins encoded by many of these genes won’t be possible based on fluorescent protein “knock-in,” but may be enabled by estradiol-titratable overexpression at the minimal detectable level. It appears likely that not only in *P. patens* protonemata but also other plant tissues endogenous expression levels may often be insufficient for microscopic imaging of fluorescent fusion proteins.

## Conclusion

Based on fluorescent protein-tagging under the best achievable conditions the distribution patterns of a ROP GTPase and a ROP-GEF during tip growth were reliably established. In apical cells of *P. patens* protonemata PpROP1 (estradiol-titratable expression at minimal detectable level) and PpROP-GEF4 (genomic “knock-in”) co-localize within a small plasma membrane domain at the extreme apex. PpROP1 is associated with a larger region of the plasma membrane at the tip, which substantially extends beyond the apical domain of co-localization. Together with the observation that the two proteins interact with each other in yeast two-hybrid assays, these findings suggest that PpROP1 may be activated by PpROP-GEF4 within the small apical plasma membrane domain of co-localization. This is consistent with a previously proposed model (Kost, 2008) predicting that PpROP1 inactivation may occur at lateral plasma membrane regions flanking the apical domain of co-localization with PpROP-GEF4. Furthermore, PpROP1 was also found to accumulate at the plasma membrane underlying the callose-enriched cross wall between apical and subapical protonemal cells, indicating that at this location PpROP1 possibly promotes callose synthesis, similar to related small GTPase in other cell types (Qadota et al., 1996; Hong et al., 2001). Interestingly, although RNA-Seq data suggest that with regards to transcript level *PpROP1* ranks among the top 80% of all genes active in *P. patens* protonemata, a YFP::PpROP1 fusion protein expressed at essentially endogenous levels in genomic “knock-in” protonemata was not detectable by confocal imaging. To circumvent this problem, we have shown that estradiol-titratable expression at the minimal detectable level constitutes an important alternative approach optimally suited to investigate the intracellular localization of YFP::PpROP1, and of other fluorescent fusion proteins in *P. patens* protonemata as well as in other plant tissues, which display endogenous expression levels insufficient for microscopic imaging.

## Author Contributions

ALB and BK designed the experiments, analyzed the data, and wrote the manuscript. ALB, SS, and MN performed the experiments. P-FP and SAR provided and analyzed the RNA-Seq data.

## Conflict of Interest Statement

The authors declare that the research was conducted in the absence of any commercial or financial relationships that could be construed as a potential conflict of interest.

## Footnotes

1 http://labs.biology.ucsd.edu/estelle/moss2.html
