# Supplementary material for: Analysis of the Localization of Fluorescent PpROP1 and PpROP-GEF4 Fusion Proteins in Moss Protonemata Based on Genomic “Knock-In” and Estradiol-Titratable Expression
Source: Front Plant Sci. 2019 Apr 12;10:456. doi: 10.3389/fpls.2019.00456 (PMC6473103; doi:10.3389/fpls.2019.00456)
Supplement: Supplementary file 1 [file Data_Sheet_1.PDF]

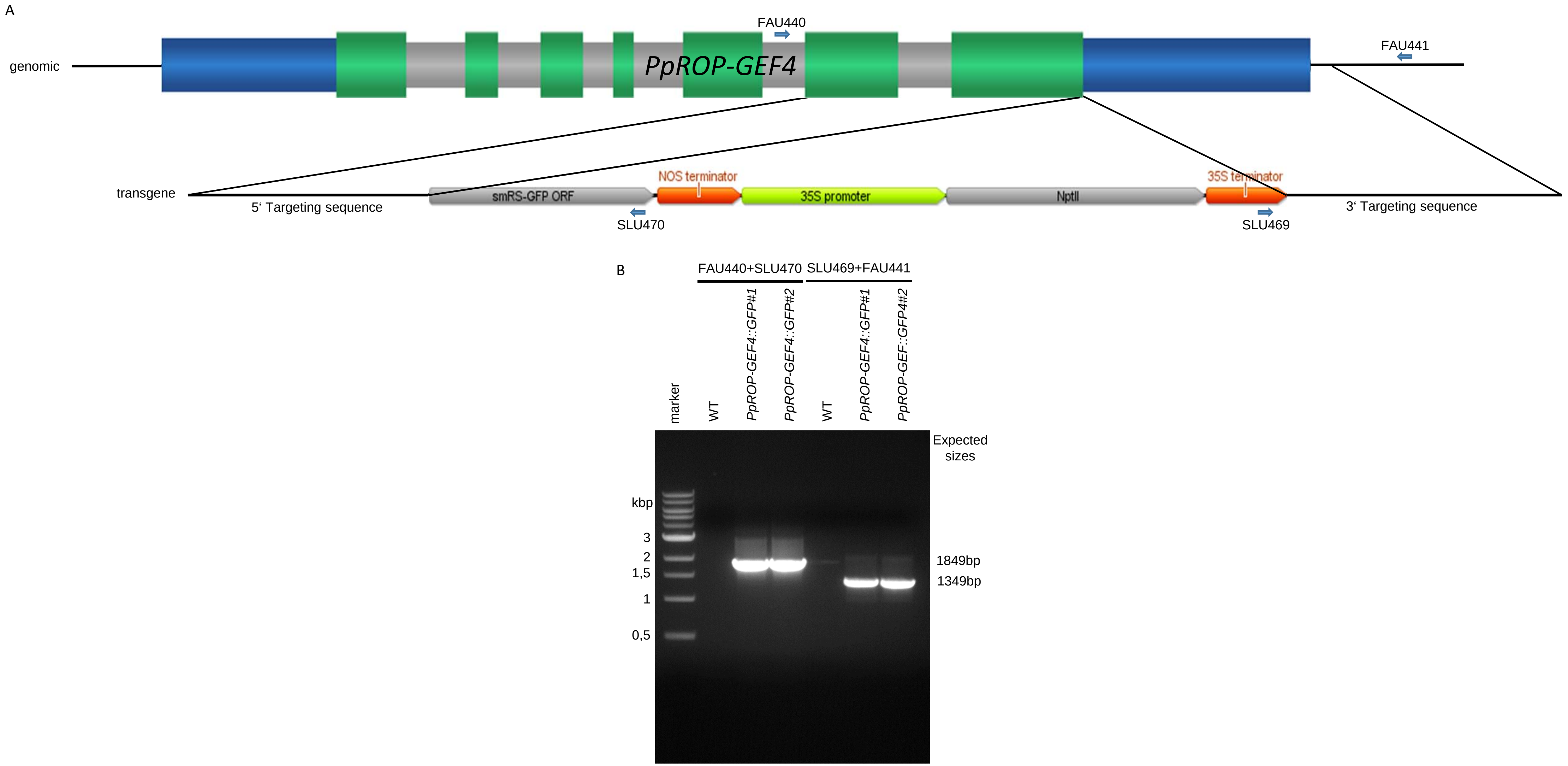

Figure S1: Genotyping of the *PpROP-GEF4::GFP#1* and *#2* "knock-in" lines. **(A)** Schematic representation of the *PpROP-GEF4* gene, of the transgene inserted into this gene as well as of the 5' and 3' targeting sequences flanking the insertion site. Arrows: primers used for PCR-based genotyping. **(B)** Ethidium bromide stained DNA gel showing PCR fragments spanning the transgene/genome junctions at both the 5' (FAU440/SLU470) and 3' (SLU469/FAU441) ends of the inserted transgene. These fragments displayed the expected sizes (1849bp and 1349bp, respectively) and could specifically be amplified from *PpROP-GEF4::GFP#1* and *#2*, but not from WT, genomic DNA.

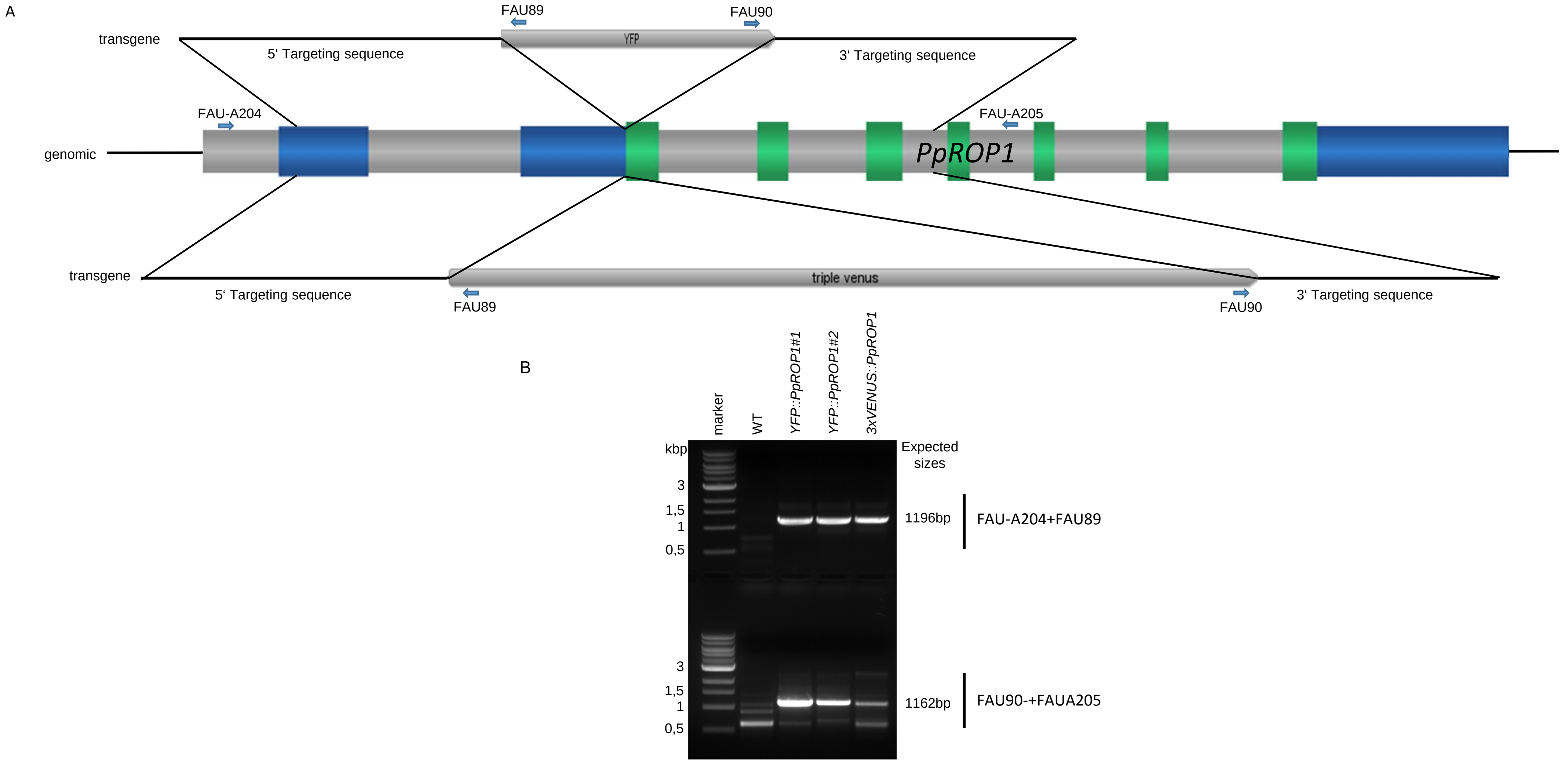

Figure S2: Genotyping of the *YFP::PpROP1#1* and *#2* as well as of the *3xVENUS::PpROP1* "knock-in" lines. **(A)** Schematic representation of the *PpROP1* gene, of the transgenes inserted into this gene as well as of the 5' and 3' targeting sequences flanking the insertion site. Arrows: primers used for PCR-based genotyping. **(B)** Ethidium bromide stained DNA gel showing PCR fragments spanning the transgene/genome junctions at both the 5' (FAU-A204/FAU89) and 3' (FAU90/FAU-A205) ends of the inserted transgene. These fragments displayed the expected sizes (1196bp and 1162bp, respectively) and could specifically be amplified from *YFP::PpROP1#1* and *#2* or *3xVENUS::PpROP1*, but not from WT, genomic DNA.

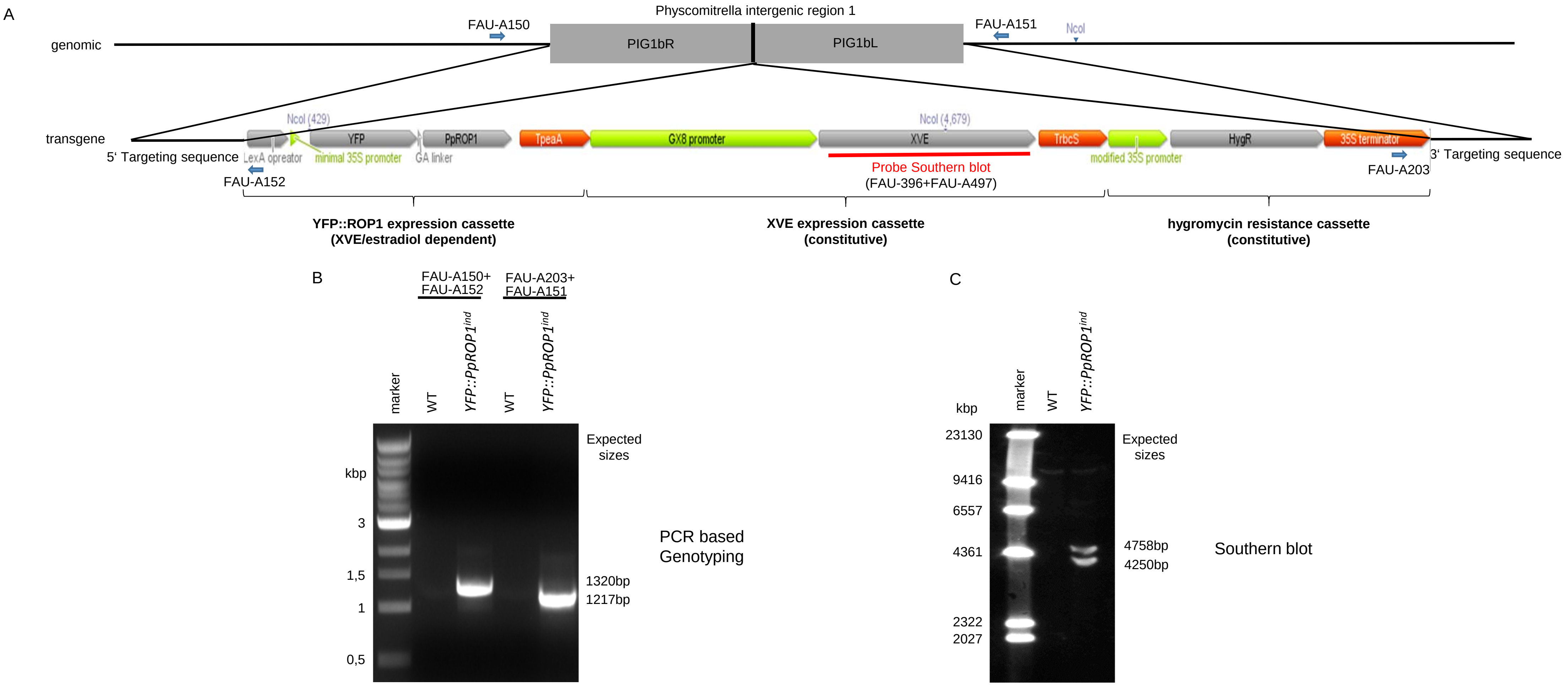

Figure S3: Genotyping of the *YFP::PpROP1^{ind}* line enabling estradiol-inducible YFP::PpROP1 expression. **(A)** Schematic representation of the Physcomitrella intergenic region 1 (PIG1), of the transgene inserted into this region as well as of the PIG1bR and PIG1bL targeting sequences flanking the insertion site. Arrows: primers used for PCR-based genotyping. **(B)** Ethidium bromide stained DNA gel showing PCR fragments spanning the transgene/genome junctions at both the 5' (FAU-A150/FAU-A152) and 3' (FAU-A203/FAU-A151) ends of the inserted transgene. These fragments displayed the expected sizes (1320bp and 1217bp, respectively) and could specifically be amplified from *YFP::PpROP1^{ind}*, but not from WT, genomic DNA. **(C)** Southern blot showing genomic WT and *YFP::PpROP1^{ind}* DNA restricted by NcoI and blotted onto a nylon membrane after separation by agarose gel electrophoresis. The membrane was hybridized with a probe corresponding to the XVE coding sequence as indicated in (A) (chemoluminescence detection). All relevant NcoI sites (2x transgene, 1x 3' genomic flanking region) are also indicated in (A). The probe specifically hybridized with two fragments with the expected sizes (4758bp and 4250bp) of *YFP::PpROP1^{ind}*, but not of WT, genomic DNA, which demonstrates single copy transgene insertion.

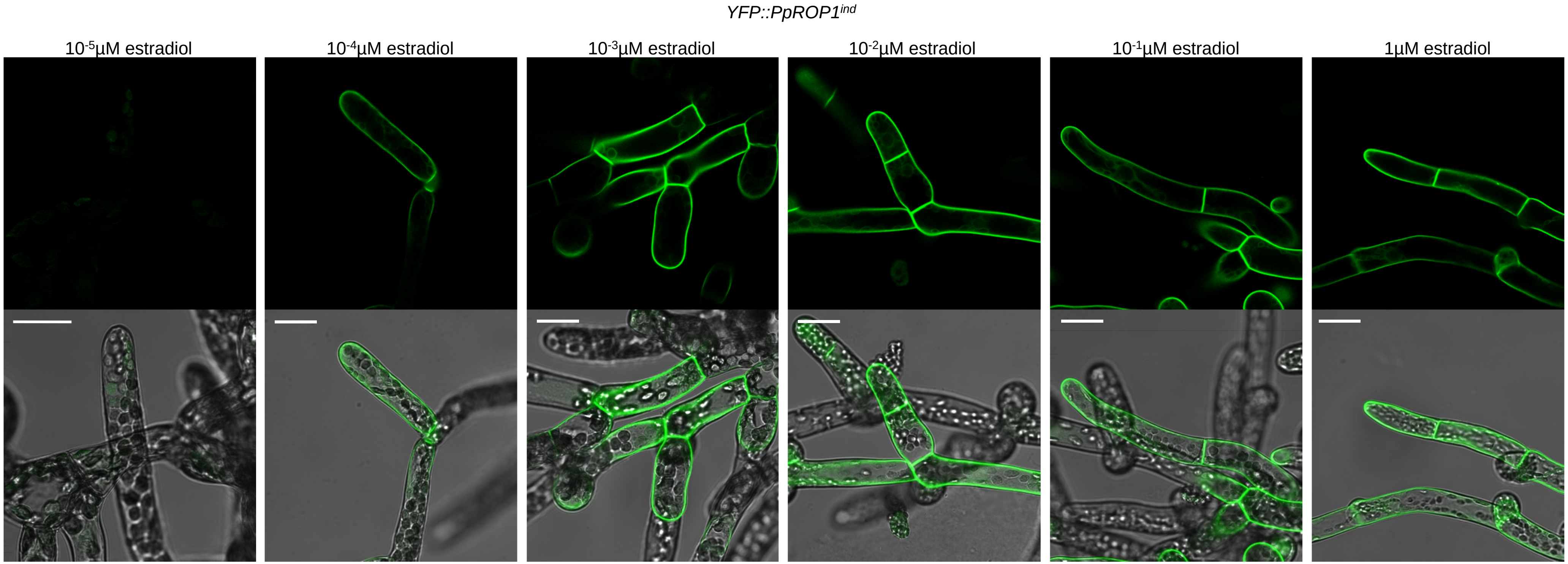

Figure S4: Analysis of the intracellular distribution of YFP::PpROP1 expressed at different levels in apical cells of *YFP::PpROP1*^{ind} protonemata induced with estradiol at concentrations ranging from 10^{-5}µM to 1µM. Upper panel: medial confocal optical sections through the tips of *YFP::PpROP1*^{ind} protonemal filaments grown in the presence of estradiol at the indicated concentrations. Only background chlorophyll autofluorescence was visible after induction with 10^{-5}M. Lower panel: the fluorescence images shown in the upper panel overlaid onto corresponding transmitted light reference images (differential interference contrast, DIC). Scale bars = 40µm.
