# Supplementary material for: Analysis of the Localization of Fluorescent PpROP1 and PpROP-GEF4 Fusion Proteins in Moss Protonemata Based on Genomic “Knock-In” and Estradiol-Titratable Expression
Source: Front Plant Sci. 2019 Apr 12;10:456. doi: 10.3389/fpls.2019.00456 (PMC6473103; doi:10.3389/fpls.2019.00456)
Supplement: Supplementary file 2 [file Data_Sheet_2.PDF]

Table S1: PCR primers used for genotyping, molecular cloning and Southern blot probe generation

name	purpose	description
FAU-A150	Genotyping *YFP::PpROP1* ^{ind}	outside PIG1bR for
FAU-A152	Genotyping *YFP::PpROP1* ^{ind}	inside PGX8 rev
FAU-A203	Genotyping *YFP::PpROP1* ^{ind}	inside PGX8 for
FAU-A151	Genotyping *YFP::PpROP1* ^{ind}	outside PIG1bL rev
SLU469	Genotyping *PpROP-GEF4::GFP*	35S terminator for
SLU470	Genotyping *PpROP-GEF4::GFP*	smRS-GFP rev
FAU440	Genotyping *PpROP-GEF4::GFP*	outside 5' targetting sequence of gef4-gfp for
FAU441	Genotyping *PpROP-GEF4::GFP*	outside 3' targetting sequence of gef4-gfp rev
FAU-A204	Genotyping *YFP::PpROP1* and *3xVENUS::PpROP1*	outside 5' targeting sequence of yfp-rop1 for
FAU89	Genotyping *YFP::PpROP1* and *3xVENUS::PpROP1*	YFP rev
FAU-A205	Genotyping *YFP::PpROP1* and *3xVENUS::PpROP1*	outside 3' targeting sequence of yfp-rop1 rev
FAU90	Genotyping *YFP::PpROP1* and *3xVENUS::PpROP1*	YFP for
FAU88	QPCR	reference gene (Ubiquitin conjugation enzyme) for
FAU28	QPCR	reference gene (Ubiquitin conjugation enzyme) rev
SLU382	QPCR	reference gene (actin 5) for
SLU383	QPCR	reference gene (actin 5) rev
FAU-A67	QPCR	endogeneous PpROP1 for
FAU-A69	QPCR	endogeneous PpROP rev
FAU-B933	QPCR	inducible PpROP1 for
FAU-B934	QPCR	inducible PpROP1 rev
SLU415	QPCR	PpROP-GEF4 for (for level in WT)
SLU416	QPCR	PpROP-GEF4 rev (for level in WT)
FAU-C28	QPCR	PpROP-GEF4 for (for level in *PpROP-GEF4::GFP*)
FAU-C29	QPCR	PpROP-GEF4 rev (for level in *PpROP-GEF4::GFP*)
SLU440	cloning *PpROP-GEF4::GFP*	3' targetting sequence for + NotI
SLU441	cloning *PpROP-GEF4::GFP*	3' targetting sequence rev + XhoI
SLU438	cloning *PpROP-GEF4::GFP*	5' targetting sequence for + BamHI
SLU444	cloning *PpROP-GEF4::GFP*	5' targetting sequence rev + BamHI
FAU23	cloning *YFP::PpROP1*	5' targetting sequence for + NdeI
FAU24	cloning *YFP::PpROP1*	5' targetting sequence rev + PacI
SLU433	cloning *YFP::PpROP1*	YFP for + PacI
SLU434	cloning *YFP::PpROP1*	YFP rev + PacI
FAU81	cloning *YFP::PpROP1*	3' targetting sequence for + EcoRV
FAU82	cloning *YFP::PpROP1*	3' targetting sequence rev + EcoRV
FAU-A56	cloning *3xVENUS::PpROP1*	amplification 3xVENUS for + SalI
FAU-A57	cloning *3xVENUS::PpROP1*	amplification 3xVENUS rev + XhoI
FAU-A81	cloning *3xVENUS::PpROP1*	5' targetting sequence rev + ClaI
FAU-A104	cloning *3xVENUS::PpROP1*	5' targetting sequence for + KpnI
FAU-A82	cloning *3xVENUS::PpROP1*	3' targetting sequence for + EcoRV + 2 nucleotides
FAU-A83	cloning *3xVENUS::PpROP1*	3' targetting sequence rev + NotI
SLU32	cloning *YFP::PpROP1* ^{ind}	PpROP1 cDNA for + XhoI
SLU33	cloning *YFP::PpROP1* ^{ind}	PpROP1 cDNA rev + ApaI
SLU70	cloning *YFP::PpROP1* ^{ind}	YFP-5xGA for + XhoI
SLU71	cloning *YFP::PpROP1* ^{ind}	YFP-5xGA rev + XhoI
FAU-A78	cloning *YFP::PpROP1* ^{ind}	YFP-5xGA-PpROP1 for
FAU-A79	cloning *YFP::PpROP1* ^{ind}	YFP-5xGA-PpROP1 rev
SLU445	cloning Y2H	GEF4 prone domain for + NdeI
FAU532	cloning Y2H	GEF4 prone domain rev + NdeI
FAU524	cloning Y2H	ROP1^{C194S} for + NdeI
FAU583	cloning Y2H	ROP1^{C194S} rev + NdeI
FAU-A396	probe Southern blot	XVE for
FAU-A397	probe Southern blot	XVE rev

**sequence**
GACTTGTGCCCTGAATGTAC
GTACGTCGAGGGGATGATAAT
GTGCAAGGTAAGAAGATGGAAA
AATCTGGGAATAGCTTGTTATTGT
GGAATTAGGGTTCTTATAGGGT
ATGCCATGTGTAATCCCA
GTTATGACCTTCATTCCATTACC
AATGCATATTCACTTAGCTCAAG
TGGTGAAACCACACCGTTA
GCCGTCCAGCTCGACCAGGAT
CTTGTCAGTAGCACATTCCC
TTCGTGACCGCCGCCGG
TACGGACCCTAATCCAGATGAC
CAACCCATTGCATACTTCTGAG
ACCGAGTCCAACATTCTACC
GTCCACATTAGATTCTCGCA
TGACATTTCTCGACCGGAC
TCAGGGGTACAGAACTCCTAC
CGAGGAGCTCAGGAAGTCAA
TGGATCGACTAGATCACCACT
TAGAGTGTTGGAGAGTTTGG
GAAGATGGTGGAGTTGGT
GCAGAGACAAGAAGAGAAGTGG
AGCGAATCCCAATACACATCAG
gcggccgcTTTTTCGGTTCACATTGAG
ctcgagTCTCCTAAAAGAAATCATGTCA
ggatccCTTCAGACAATTACTCAGCA
ggatccGGTCACGACCCGGAG
catatgCAAAAGGGAGTCAGTCAGTTTA
ttaattaaGACTGCGTCCCAGCT
ttaattaaATGGTGAGCAAGGGCGAGGA
ttaattaaGGCACCAGCACCAGCACCA
gatatcATGAGCACCTCCCGG
gatatcGGATCCACTGCAATGCA
gtcgacGTTCGAAATCGATAAGCTTGGATCC
ctcgagTTCGGCCGCAATGTACAGC
atcgatGACTGCGTCCCAGCT
ggtaccCAAAAGGGAGTCAGTCAGTTTA
gatatcCCATGAGCACCTCCCGG
gcggccgcGGATCCACTGCAATGCA
caaactcgagATGAGCACCTCCCGGTTC
caaagggcccTCAGAGTATGACACAG
gtttctcgagATGGTGAGCAAGGGCGAGGA
gtttctcgagGGCACCAGCACCAGCACCA
caccATGGTGAGCAAGGGCG
TCAGAGTATGACACAGTTCTTTTG
catatgGACGATGCAAGTACTATTAGC
ggatccGACTGACTTCTTCACCATGT
catatgATGAGCACCTCCCGG
gaattcTCAGAGTATGACAGAGTTCTTTT
ATGAAAGCGTTAACGGCCA
TCAGACTGTGGCAGGGAA
